# The benefit and risk of adding PD-1/PD-L1 inhibitors plus anti-VEGF drugs to transarterial chemoembolisation for unresectable, non-metastatic hepatocellular carcinoma: a pooled analysis of four RCTs

**DOI:** 10.3389/fmed.2026.1792746

**Published:** 2026-05-25

**Authors:** Tonggang Zhou, Chunlin Yu, Baoliang Zhong, Linqing Wang, Xiaoqing Zeng, Yan Wang

**Affiliations:** 1 Department of Interventional and Vascular Surgery, Ganzhou Hospital-Nanfang Hospital, Southern Medical University, Ganzhou, Jiangxi, China; 2 Interventional Medicine Ganzhou City Key Laboratory, Ganzhou, Jiangxi, China

**Keywords:** anti-VEGF drugs, hepatocellular carcinoma, meta-analysis, PD-1/PD-L1 inhibitors, randomized controlled trials, transarterial chemoembolisation

## Abstract

**Background:**

Transarterial chemoembolisation (TACE) is the standard treatment for unresectable, non-metastatic hepatocellular carcinoma (UR/NM HCC). The addition of PD-1/PD-L1 inhibitors (PIs) plus anti-VEGF drugs (AVDs) after TACE (TPA group) has been proposed as a strategy to enhance antitumor efficacy; however, evidence regarding its survival benefit is still uncertain. To address this issue, we performed a pooled analysis of randomized controlled trials (RCTs) to determine whether this combination strategy confers additional clinical benefit over TACE alone.

**Methods:**

Six databases were systematically searched to identify eligible RCTs comparing TPA with TACE alone among individuals diagnosed with UR/NM HCC. Key endpoints consisted of overall survival (OS) and progression-free survival (PFS), whereas additional endpoints covered tumor responses, adverse events (AEs), and patient status at the data cutoff. PFS and tumor responses were assessed according to both RECIST version 1.1 and mRECIST criteria.

**Results:**

A total of 4 RCTs (CARES-005, EMERALD-1, LEAP-012, and TALENTACE) involving 1,431 patients were included. Compared with TACE alone, TPA significantly improved PFS (HR: 0.62 [0.47, 0.81], *p* = 0.0006) and objective response rate (ORR, RR: 1.44 [1.25, 1.66], *p* < 0.00001), whereas no statistically significant improvement in OS was observed (HR: 0.87 [0.71, 1.07], *p* = 0.20). Subgroup analyses demonstrated that the PFS benefit of the TPA group was consistent across nearly all predefined subgroups. However, TPA also showed an increased occurrence of grade 3–4/serious treatment-emergent AEs (TEAEs), grade 3–4/serious treatment-related AEs (TRAEs), and total/grade 3–4 immune-related AEs (irAEs). At the cutoff, more patients in the TPA group discontinued treatment due to AEs, whereas fewer discontinued due to disease progression.

**Conclusion:**

In patients with UR/NM HCC, TPA significantly improves PFS and ORR but does not yet demonstrate a significant OS benefit, and it is associated with increased grade 3–4 AEs.

**Systematic review registration:**

https://www.crd.york.ac.uk/PROSPERO/view/CRD420261290301, CRD420261290301.

## Introduction

Hepatocellular carcinoma (HCC) continues to rank among the most fatal malignancies globally and is frequently diagnosed during a non-metastatic but unresectable stage, when curative options are no longer feasible (1). Transarterial chemoembolisation (TACE) serves as the standard therapeutic approach applied to this population and is recommended by international guidelines for intermediate-stage and selected locally advanced disease (2). TACE provides local tumor control through targeted chemotherapy and ischemia-induced necrosis, but it is rarely curative and often requires repeated sessions (3). Tumor recurrence, treatment refractoriness, and progressive liver dysfunction frequently occur, thereby limiting long-term survival and highlighting the need for more effective post-TACE strategies (4).

The emergence of immunotherapy and anti–vascular endothelial growth factor (anti-VEGF) agents has reshaped current therapeutic paradigms in HCC (5). Combination regimens of PD-1/PD-L1 inhibitors (PIs) and anti-VEGF drugs (AVDs) have demonstrated superior survival outcomes in advanced disease, providing a strong biological and clinical rationale for earlier integration with locoregional therapies (6). TACE can promote tumor antigen release and immunogenic cell death, potentially enhancing immune responses, but it also induces hypoxia-driven VEGF upregulation and immunosuppressive changes in the tumor microenvironment that may facilitate tumor progression (7). Therefore, combining TACE with PIs plus AVDs (TPA) may synergistically enhance antitumor efficacy by augmenting immune activation while counteracting angiogenesis and post-embolization tumor escape mechanisms.

Several recent randomized controlled trials (RCTs) have evaluated this triple-modality approach among individuals diagnosed with unresectable, non-metastatic HCC (UR/NM HCC) (8, 9). Although most trials consistently reported improvements in progression-free survival (PFS) and tumor response, overall survival (OS) benefits remain inconclusive. In addition, TPA is associated with higher rates of adverse events (AEs), which may compromise treatment adherence and liver function in cirrhotic patients. To date, evidence synthesized exclusively from RCTs remains limited in determining whether the addition of PIs plus AVDs after TACE provides sufficient clinical benefit to justify routine use in this population. Therefore, we performed a meta-analysis of RCTs comparing TPA with TACE alone in UR/NM HCC to comprehensively assess the balance between efficacy and safety and to inform optimal treatment strategies.

## Materials and methods

### Search strategy

The search strategy included terms such as “transarterial chemoembolization,” “PD-1/PD-L1 inhibitors,” “hepatocellular carcinoma,” and “randomized.” Records were retrieved from PubMed, ScienceDirect, the Cochrane Library, Scopus, EMBASE, and Web of Science from inception to 27 December 2025. The full search strategy is presented in Supplementary Table S1.

### Selection criteria

The research question was defined using the PICOS framework as follows:

(1) Participants (P): Patients with UR/NM HCC;
(2) Intervention (I) and Control (C): TPA versus TACE alone;
(3) Outcomes (O): Key endpoints consisted of OS and PFS, while additional endpoints covered tumor responses, AEs, and patient status at the data cutoff.
(4) Study design (S): RCTs.

Exclusion criteria:

(1) Retrospective studies, single-arm studies, case reports, narrative reviews, meta-analyses, and animal studies;
(2) Studies without sufficient data.

### Data extraction

Using a standardized data extraction form, two investigators independently extracted trial characteristics, patient baseline features (including age and ECOG PS), survival outcomes (PFS, OS), tumor response, and AEs. Missing data were obtained by contacting the study authors, and any discrepancies were resolved through discussion until consensus was achieved.

### Outcome assessments

All outcome definitions were prespecified. OS was defined as time from randomisation to death from any cause. PFS was defined as time from randomisation to first documented disease progression (per RECIST 1.1 or mRECIST) or death (10, 11). PFS and OS were examined across subgroups defined by age, sex, ECOG PS, geographic region, aetiology of HCC, Barcelona Clinic liver cancer (BCLC) stage, portal vein invasion, Child-Pugh score at screening, baseline tumor burden, baseline albumin-bilirubin grade, PD-1PD-L1 inhibitor type and Anti-VEGF drug type. PFS rates (PFSR) and OS rates (OSR) were evaluated at quarterly intervals from 3 to 36 months. PFS and tumor responses were assessed using both RECIST version 1.1 and mRECIST criteria (10, 11).

### Quality assessment

Methodological quality was assessed by two reviewers independently using two established instruments: the Cochrane Risk of Bias tool and the Jadad scale. The Jadad scale, which assigns scores from 1 to 7, considers studies scoring 5–7 as high quality (12, 13). The GRADE approach was used to evaluate the overall strength of evidence, rating the certainty of results on four levels, from very low to high (14).

### Statistical analysis

Meta-analyses were conducted with Review Manager 5.3 and STATA 12.0. Hazard ratios (HRs) summarized time-to-event outcomes, mean differences (MDs) represented continuous outcomes, and risk ratios (RRs) described categorical variables. The choice of statistical model was based on heterogeneity: low heterogeneity (*I^2^* < 50% or *p* > 0.1) favored a fixed-effect model, whereas high heterogeneity led to the use of a random-effects model. Significance was determined by a two-tailed *p* value < 0.05. Possible publication bias was assessed through visual examination of funnel plot symmetry. Sensitivity analyses were performed by sequentially excluding each individual trial to assess the influence of any single study on the pooled estimates.

## Results

### Search results

**Figure 1:**
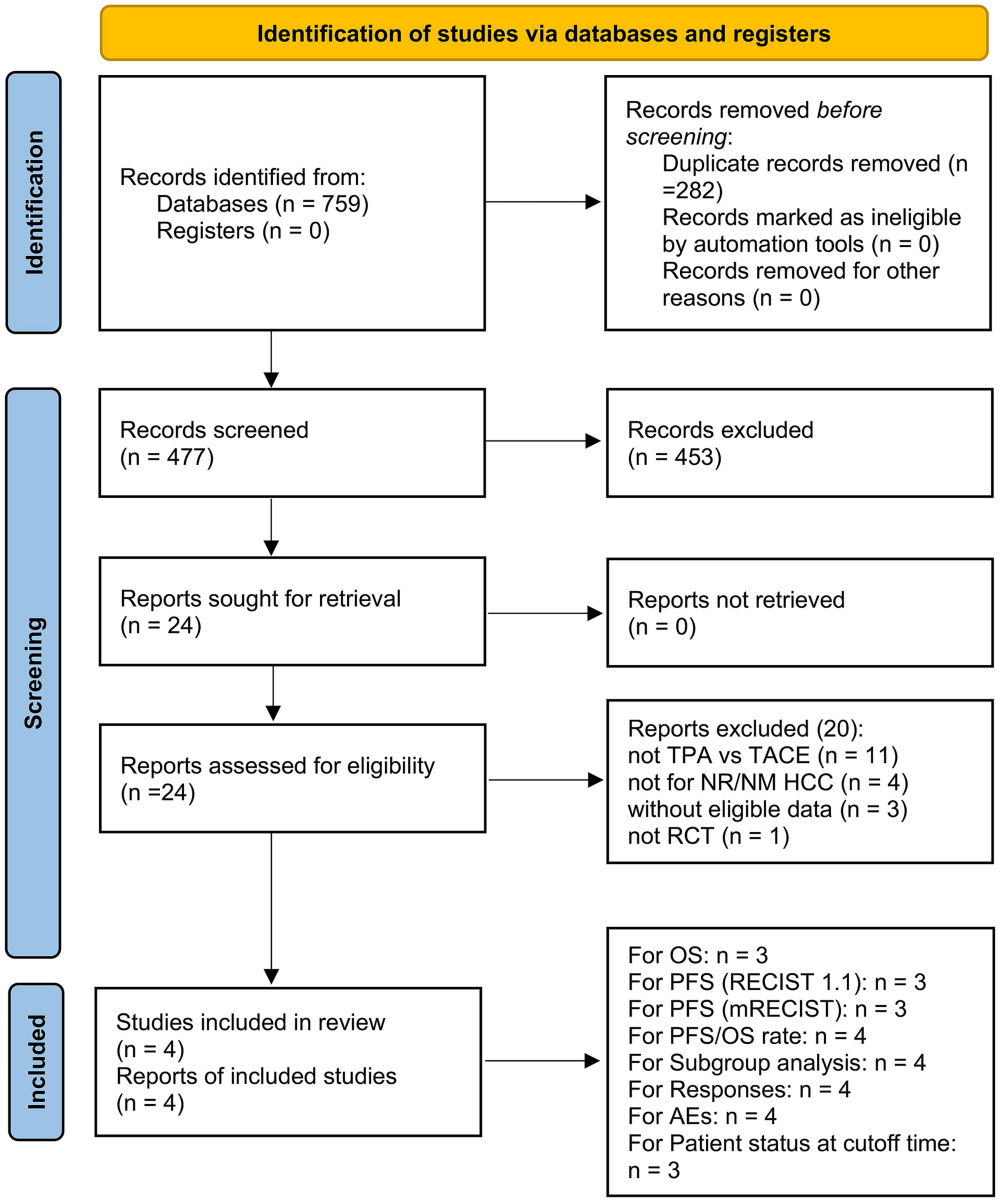
Flow diagram of study selection, screening, and eligibility assessment.

Of 477 studies screened, four RCTs (CARES-005, EMERALD-1, LEAP-012, and TALENTACE), including 1,431 participants, met the inclusion criteria (Figure 1) (8, 9, 15, 16). Three trials—EMERALD-1, LEAP-012, and TALENTACE—were international, multicenter studies, whereas CARES-005 was conducted in China. All four RCTs were of high methodological quality (Supplementary Figure S1; Supplementary Table S2). Key study and participant characteristics are presented in Table 1. Using the GRADE methodology, overall certainty regarding the findings was rated medium – high (Supplementary Table S3).

**Table 1:** Baseline characteristics of the included studies.

Study	Year	Registration ID	Phase	Groups	Patients	Sex (M/F)	Age (Mean, year)	ECOG PS		BCLC stage			PI type	AVD type	Follow up (months)
								0	1	A	B	C			
CARES-005 (15)	2025	NCT04559607	Phase 2	TPA	100	87/13	58.5	56	44	14	44	42	Camrelizumab	Rivoceranib	14.3
				TACE	100	87/13	57.0	58	42	14	42	44			14.3
EMERALD-1 (8)	2025	NCT03778957	Phase 3	TPA	204	162/42	64.5	167	37	51	117	35	Durvalumab	Bevacizumab	27.9
				TACE	205	163/42	66.0	175	30	49	122	21			27.9
LEAP-012 (9)	2025	NCT04246177	Phase 3	TPA	237	192/45	65.0	216	21	80	135	21	Pembrolizumab	Lenvatinib	25.8
				TACE	243	206/37	66.0	213	30	68	146	29			25.4
TALENTACE (16)	2025	NCT04712643	Phase 3	TPA	171	136/35	62.0	139	31	36	100	35	Atezolizumab	Bevacizumab	26.0
				TACE	171	140/31	60.0	149	22	42	105	24			26.0

AVDs, Anti-VEGF drugs; BCLC, Barcelona Clinic Liver Cancer; ECOG PS, Eastern Cooperative Oncology Group Performance Status; M/F, Male/Female; PD-1, Programmed cell death protein 1; PD-L1, Programmed death-ligand 1; PIs, PD-1/PD-L1 inhibitors; TACE, Transarterial chemoembolization; TPA, TACE plus PIs and AVDs; VEGF, Vascular endothelial growth factor.

### Patient status at cutoff time

At the cutoff, a similar number of patients in each group were ongoing/completed treatment (RR: 1.29 [0.84, 1.96], *p* = 0.24). Patients in the TACE arm exhibited an elevated exclusion rate, primarily due to disease progression (RR: 0.74 [0.58, 0.94], *p* = 0.01; overall RR: 0.92 [0.86, 0.98], *p* = 0.007). Conversely, treatment discontinuations due to AEs were more frequent in the TPA group (RR: 3.12 [2.08, 4.67], *p* < 0.00001) (Supplementary Table S4).

### Survival

**Figure 2:**
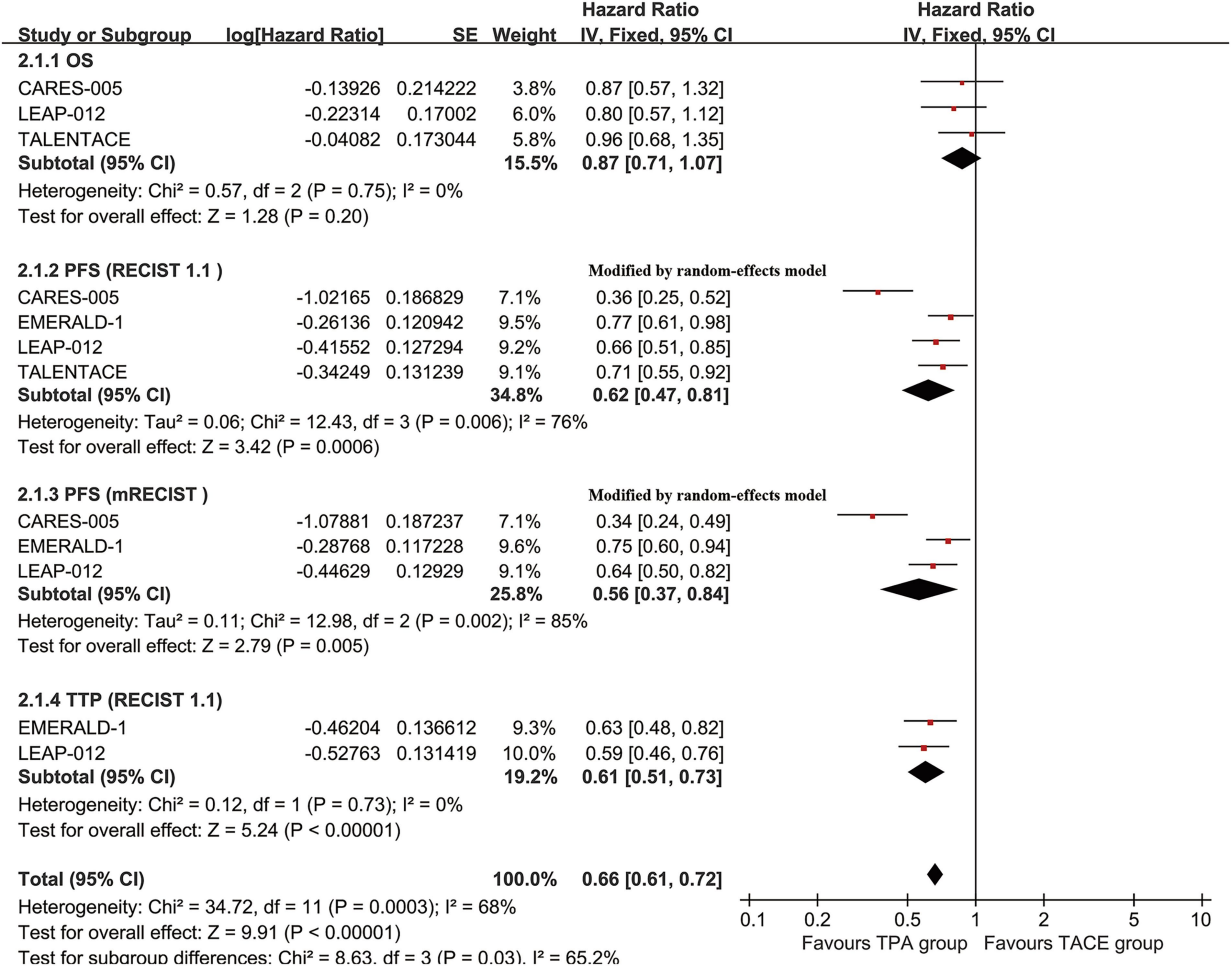
Forest plots illustrating OS, PFS (RECIST 1.1), PFS (mRECIST), and TTP (RECIST 1.1) outcomes for TPA versus TACE.

**Figure 3:**
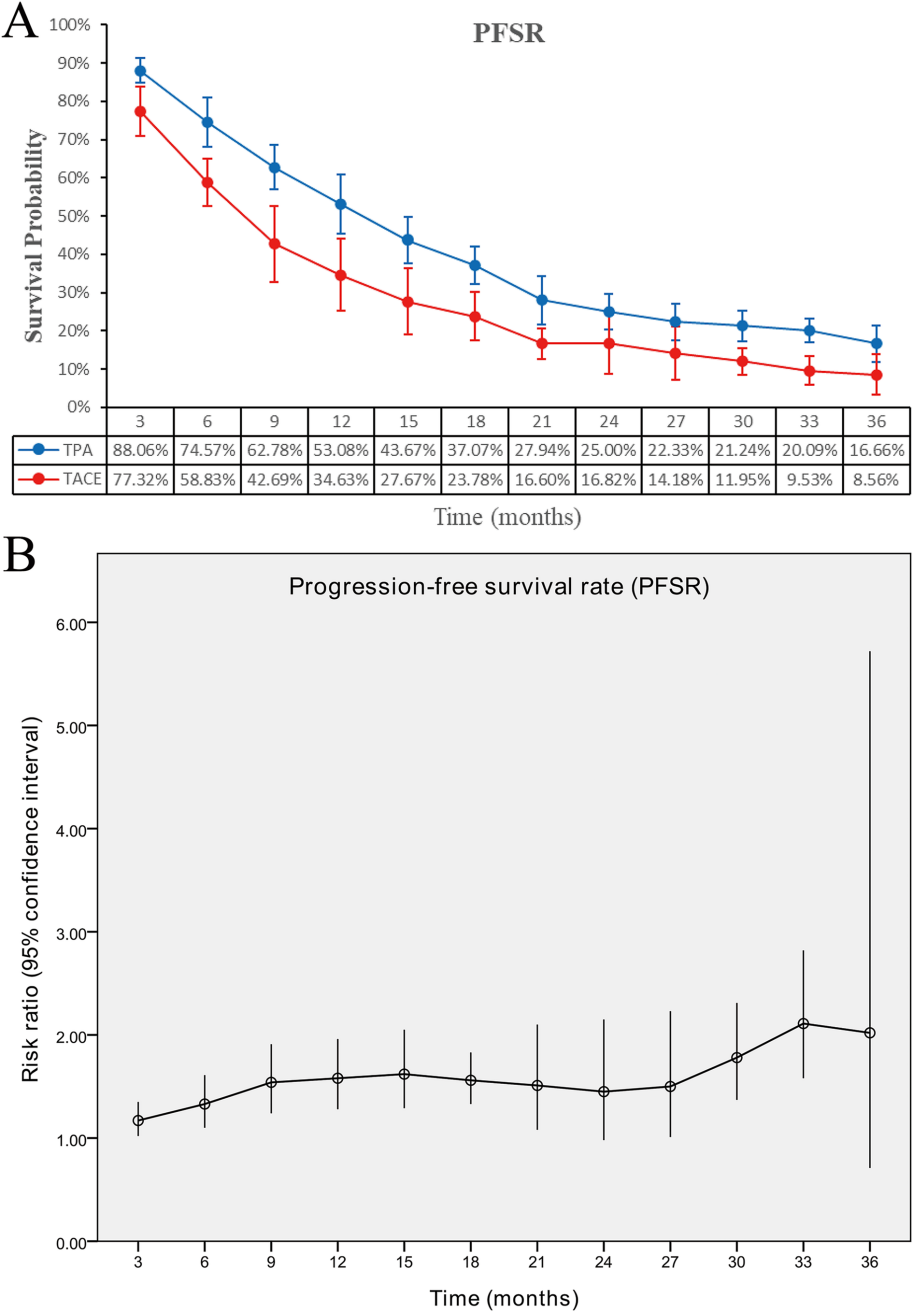
Comparison of progression-free survival rates (PFSR) between TPA and TACE. **(A)** PFSR from 3 to 36 months; **(B)** corresponding risk ratios.

Using RECIST version 1.1, the TPA group showed superior PFS compared with the TACE group (HR: 0.62 [0.47, 0.81], *p* = 0.0006) (Figure 2). PFSR generally favored TPA over 3–36 months, with statistically significant benefit observed at 3–21 and 27–33 months (Figure 3; Supplementary Figure S2). Similarly, TTP was longer in the TPA group (HR: 0.61 [0.51, 0.73], *p* < 0.00001) (Figure 2). Consistent results were obtained using mRECIST, with PFS also favoring TPA (HR: 0.56 [0.37, 0.84], *p* = 0.005) (Figure 2).

**Figure 4:**
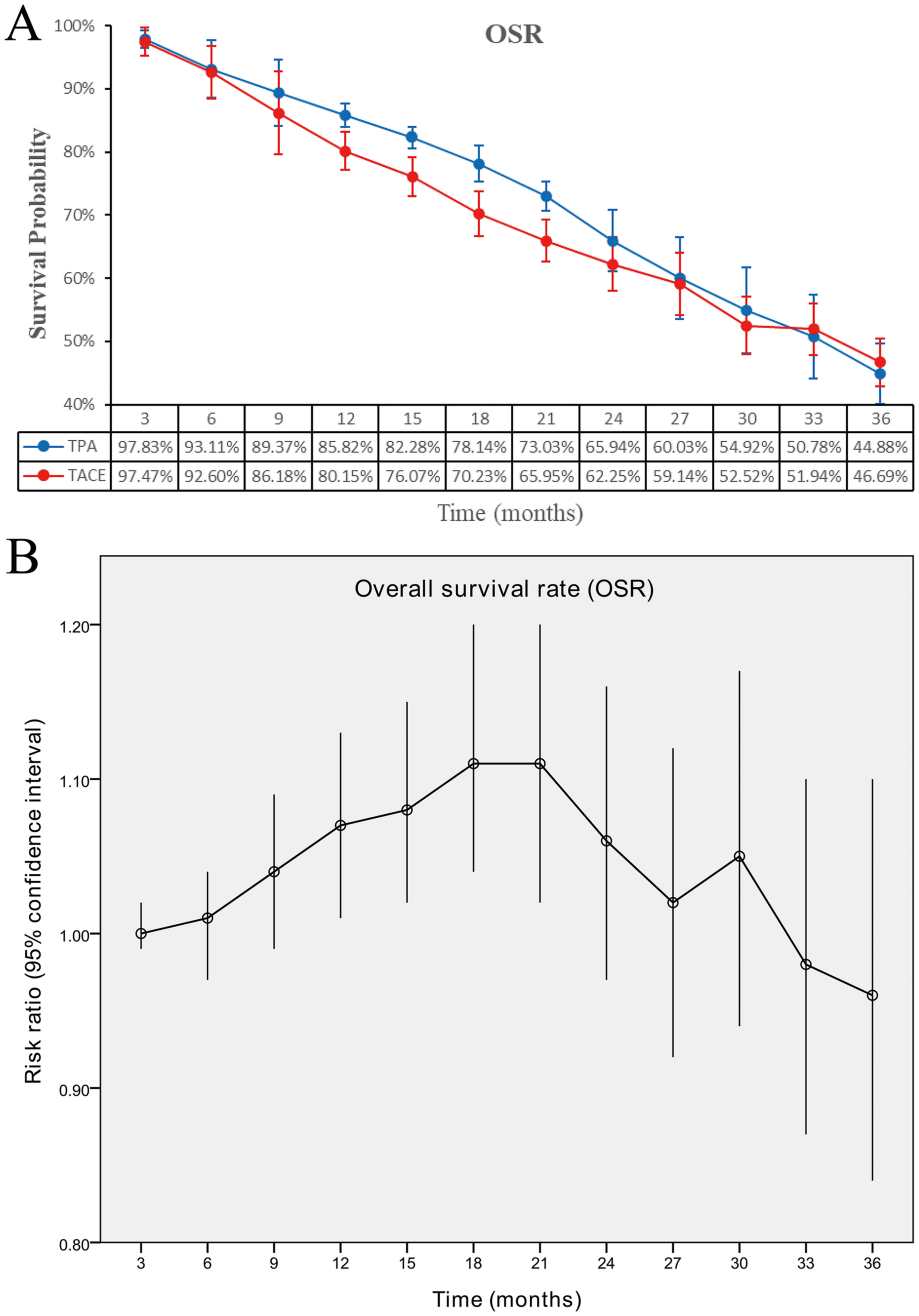
Comparison of overall survival rate (OSR) between TPA and TACE. **(A)** OSR from 3 to 36 months; **(B)** corresponding risk ratios.

OS tended to favor the TPA group, yet the observed difference failed to achieve statistical significance (HR: 0.87 [0.71, 1.07], *p* = 0.20) (Figure 2). OSR was similar between groups at 3–9 and 21–36 months, but demonstrated a notable increase in the TPA arm at 12–18 months (Figure 4; Supplementary Figure S3).

### Subgroup analysis of survival

PFS favored the TPA group across nearly all subgroups (as detailed in outcome assessments) and was consistent with the overall population. In contrast, PFS was comparable between groups in the subgroup of patients with Child-Pugh class A = 6 or hepatitis C etiology (Table 2).

**Table 2:** Subgroup analysis of progression-free survival.

Subgroups	Included studies	Progression-free survival			
		Patients	HR (95% CI)	*I^2^*	*p*
All patients	4	1,431	0.62 [0.47, 0.81]	76%	0.0006
Age					
< 65 years	4	765	0.61 [0.44, 0.85]	71%	0.003
> 65 years	4	666	0.67 [0.55, 0.81]	17%	< 0.0001
Sex					
Female	4	258	0.78 [0.57, 1.05]	0%	0.1
Male	4	1,173	0.59 [0.44, 0.80]	75%	0.0005
ECOG PS					
0	4	1,173	0.65 [0.57, 0.76]	48%	< 0.00001
1	4	257	0.63 [0.33, 1.23]	79%	0.17
Geographic region					
Japan	2	64	0.78 [0.43, 1.40]	0%	0.4
Asia (not including Japan)	4	994	0.59 [0.43, 0.80]	74%	0.0008
Aetiology of liver disease					
Hepatitis B	4	883	0.61 [0.46, 0.81]	62%	0.0005
Hepatitis C	3	190	0.84 [0.59, 1.20]	15%	0.33
Nonviral	4	365	0.61 [0.47, 0.80]	45%	0.0003
Barcelona Clinic liver cancer stage					
A	3	326	0.78 [0.58, 1.03]	0%	0.08
B	3	725	0.68 [0.57, 0.82]	10%	< 0.0001
C	4	261	0.66 [0.40, 1.09]	65%	0.1
Portal vein invasion					
Vp1/Vp2	3	63	0.70 [0.36, 1.36]	21%	0.29
None	3	888	0.59 [0.39, 0.88]	80%	0.01
Child-Pugh score at screening					
Class A = 5	2	694	0.63 [0.52, 0.77]	4%	< 0.00001
Class A = 6	2	128	1.08 [0.71, 1.64]	0%	0.73
Baseline tumour burden (criteria) ^a^					
6/7 or less	3	448	0.70 [0.55, 0.89]	0%	0.004
> 6/7	4	967	0.58 [0.41, 0.82]	79%	0.002
Baseline albumin-bilirubin grade					
1	2	588	0.65 [0.53, 0.80]	25%	< 0.0001
2 or more	2	300	0.79 [0.60, 1.03]	0%	0.08
PD-1/PD-L1 inhibitor type					
Durvalumab	1	409	0.77 [0.61, 0.98]	—	0.03
Pembrolizumab	1	480	0.66 [0.51, 0.85]	—	0.001
Atezolizumab	1	342	0.71 [0.55, 0.92]	—	0.009
Camrelizumab	1	200	0.36 [0.25, 0.52]	—	< 0.00001
Anti-VEGF drug type					
Bevacizumab	2	751	0.74 [0.62, 0.88]	0%	0.0008
Lenvatinib	1	480	0.66 [0.51, 0.85]	—	0.001
Rivoceranib	1	200	0.36 [0.25, 0.52]	—	< 0.00001

BCLC, Barcelona Clinic Liver Cancer; CI, Confidence interval; ECOG PS, Eastern Cooperative Oncology Group Performance Status; HR, Hazard ratio; I^2^, I-squared statistic; P, Probability; PD-1, Programmed cell death protein 1; PD-L1, Programmed death-ligand 1; PFS, Progression-free survival; RECIST, Response Evaluation Criteria in Solid Tumors; VEGF, Vascular endothelial growth factor.

^a^ CARES-005 and EMERALD-1 trials used the up-to-7 criteria as the cutoff to stratify tumor burden, whereas the LEAP-012 and TALENTACE trials used the up-to-6 criteria as the cutoff for tumor burden stratification (according to RECIST version 1.1).

### Responses

**Figure 5:**
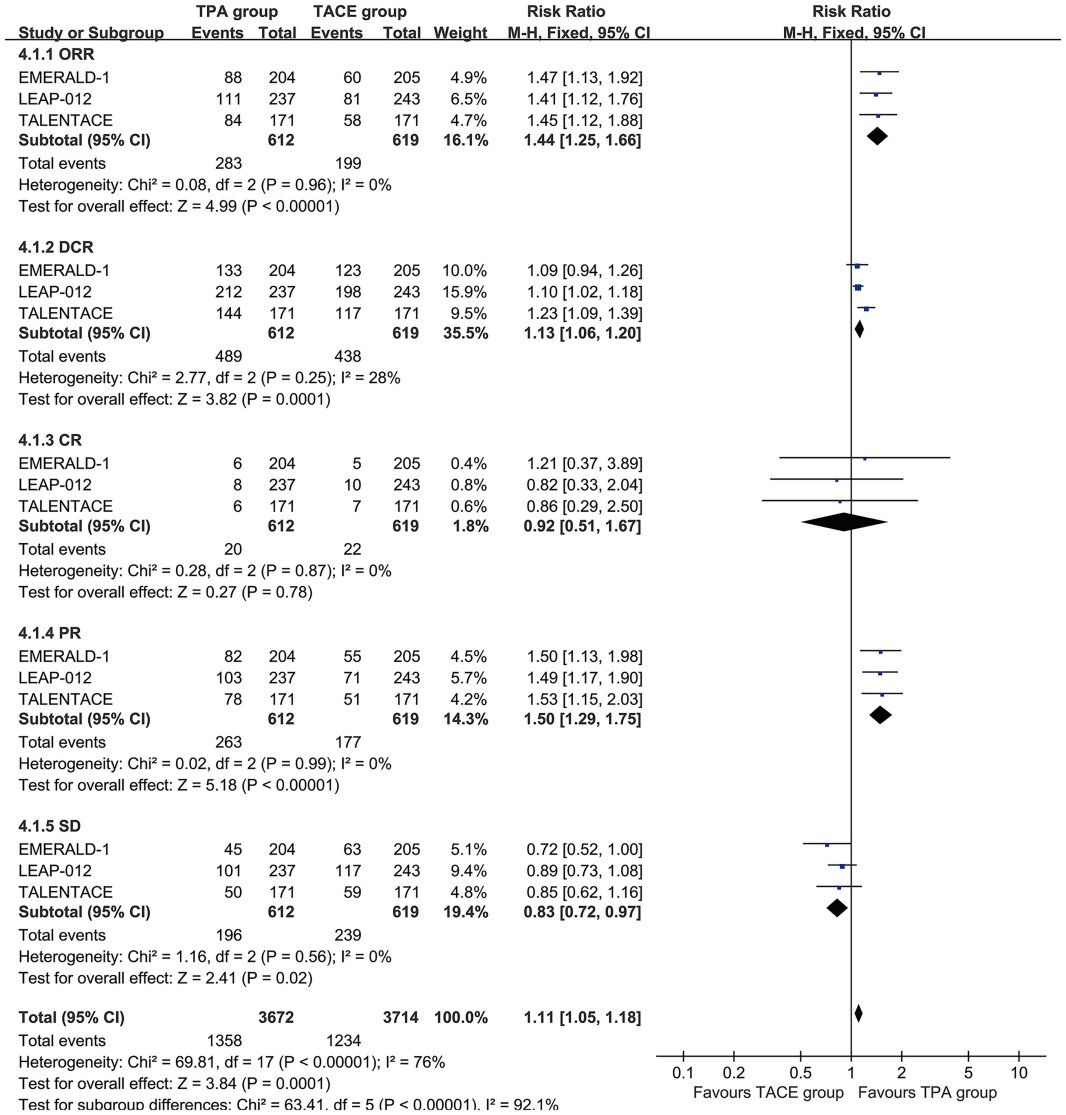
Forest plots illustrating tumor response rates based on RECIST version 1.1 for TPA versus TACE.

According to RECIST version 1.1, the objective response rate (ORR), disease control rate (DCR), and partial response (PR) were higher in the TPA group. The stable disease (SD) and progressive disease (PD) were higher in the TACE group. The complete response (CR) was similar across both arms (Table 3; Figure 5). The duration of response was also longer in the TPA group (Supplementary Figure S4).

**Table 3:** Summary of objective response outcomes according to RECIST version 1.1 and mRECIST.

Responses	TPA		TACE		Risk ratio [95% CI]	*I^2^*	*p*
	Event/Total	%	Event/total	%			
According to RECIST version 1.1							
ORR	283/612	46.24%	199/619	32.15%	1.44 [1.25, 1.66]	0%	< 0.00001
DCR	489/612	79.90%	438/619	70.76%	1.13 [1.06, 1.20]	28%	0.0001
CR	20/612	3.27%	22/619	3.55%	0.92 [0.51, 1.67]	0%	0.78
PR	263/612	42.97%	177/619	28.59%	1.50 [1.29, 1.75]	0%	< 0.00001
SD	196/612	32.03%	239/619	38.61%	0.83 [0.72, 0.97]	0%	0.02
PD	107/612	17.48%	159/619	25.69%	0.61 [0.38, 0.97]	73%	0.04
According to mRECIST							
ORR	369/508	72.64%	264/514	51.36%	1.47 [1.16, 1.87]	81%	0.002
DCR	458/508	90.16%	391/514	76.07%	1.21 [1.07, 1.36]	75%	0.002
CR	216/508	42.52%	134/514	26.07%	1.64 [1.38, 1.94]	47%	< 0.00001
PR	153/508	30.12%	130/514	25.29%	1.22 [0.86, 1.72]	65%	0.26
SD	89/508	17.52%	127/514	24.71%	0.71 [0.56, 0.90]	3%	0.005
PD	21/508	4.13%	90/514	17.51%	0.24 [0.15, 0.37]	0%	< 0.00001

AVDs, Anti-VEGF drugs; CI, Confidence interval; CR, Complete response; DCR, Disease control rate; I^2^, I-squared statistic; mRECIST, Modified Response Evaluation Criteria in Solid Tumors; ORR, Objective response rate; P, Probability; PD, Progressive disease; PD-1, Programmed cell death protein 1; PD-L1, Programmed death-ligand 1; PIs, PD-1/PD-L1 inhibitors; PR, Partial response; RECIST, Response Evaluation Criteria in Solid Tumors; RR, Risk ratio; SD, Stable disease; TACE, Transarterial chemoembolization; TPA, TACE plus PIs and AVDs; VEGF: Vascular endothelial growth factor.

According to mRECIST, the ORR, DCR, and CR were higher in the TPA group. The SD and PD were higher in the TACE group. The PR was similar across both arms (Table 3; Supplementary Figure S5).

### Safety

To summarize, TPA treatment demonstrated an increased occurrence of grade 3-4/serious treatment-emergent AEs (TEAEs), grade 3-4/serious treatment-related AEs (TRAEs), total/grade 3–4 immune-related AEs (irAEs), and TEAEs leading to death. In contrast, total TEAEs, total TRAEs, and TRAEs leading to death were similar between the two arms (Table 4; Supplementary Figure S6).

**Table 4:** Summary of adverse events.

Adverse events	TPA		TACE		Risk ratio [95% CI]	*I^2^*	P
	Event/total	%	Event/total	%			
TEAEs							
Total TEAEs	553/557	99.28%	591/616	95.94%	1.03 [0.99, 1.07]	83%	0.13
Grade 3–4 TEAEs	378/557	67.86%	249/616	40.42%	1.63 [1.32, 2.02]	71%	< 0.00001
Serious TEAEs	141/320	44.06%	103/373	27.61%	1.62 [1.32, 1.98]	0%	< 0.00001
TEAEs leading to discontinuation	157/557	28.19%	37/616	6.01%	4.73 [3.36, 6.65]	16%	< 0.00001
TEAEs leading to death	33/557	5.92%	22/616	3.57%	1.73 [1.03, 2.93]	0%	0.04
TRAEs							
Total TRAEs	524/557	94.08%	463/616	75.16%	1.28 [0.90, 1.83]	99%	0.17
Grade 3–4 TRAEs	311/557	55.83%	158/616	25.65%	2.28 [1.45, 3.60]	87%	0.0004
Serious TRAEs	73/320	22.81%	34/373	9.12%	2.58 [1.26, 5.28]	68%	0.01
TRAEs leading to discontinuation	83/391	21.23%	15/443	3.39%	6.11 [3.57, 10.47]	2%	< 0.00001
TRAEs leading to death	9/557	1.62%	7/616	1.14%	1.39 [0.53, 3.69]	29%	0.50
irAEs							
Total irAEs	165/391	42.20%	49/443	11.06%	3.76 [2.82, 5.02]	0%	< 0.00001
Grade 3–5 irAEs	21/237	8.86%	4/243	1.65%	5.38 [1.88, 15.45]	-	0.002

AEs, Adverse events; AVDs, Anti-VEGF drugs; CI, Confidence interval; I^2^, I-squared statistic; irAEs, Immune-related adverse events; P, Probability; PD-1, Programmed cell death protein 1; PD-L1, Programmed death-ligand 1; PIs, PD-1/PD-L1 inhibitors; RR, Risk ratio; TACE, Transarterial chemoembolization; TEAEs, Treatment-emergent adverse events; TPA, TACE plus PIs and AVDs; TRAEs, Treatment-related adverse events; VEGF, Vascular endothelial growth factor.

The TPA arm had significantly higher rates of any-grade and grade 3–4 adverse events across multiple categories, including hypertension, proteinuria, hepatic enzyme elevations (AST, ALT, GGT), hematologic abnormalities (thrombocytopenia, neutropenia, leukopenia, anemia), gastrointestinal events (diarrhea, nausea, abdominal pain), palmar-plantar erythrodysesthesia, hypothyroidism, and rash. The tables provide the complete list and comparative statistics (Table 5; Supplementary Table S5).

**Table 5:** Treatment-related adverse events (any grade, > 20% in the TPA group; grade 3–4, > 2% in the TPA group).

TRAEs	TPA		TACE		Risk ratio [95% CI]	*I^2^*	P
	Event/total	%	Event/total	%			
Any grade TRAEs							
Increased aspartate aminotransferase	192/497	38.63%	137/519	26.40%	1.50 [1.12, 2.01]	61%	0.006
Hypertension	234/651	35.94%	69/719	9.60%	3.81 [2.37, 6.14]	60%	< 0.00001
Proteinuria	234/651	35.94%	28/719	3.89%	12.99 [3.88, 43.53]	80%	< 0.0001
Post-embolisation syndrome	163/557	29.26%	209/616	33.93%	0.86 [0.72, 1.01]	0%	0.07
Hypothyroidism	112/391	28.64%	38/443	8.58%	3.18 [1.34, 7.55]	83%	0.009
Increased alanine aminotransferase	181/651	27.80%	144/719	20.03%	1.33 [1.10, 1.59]	44%	0.002
Palmar-plantar erythrodysesthesia syndrome	88/331	26.59%	4/346	1.16%	20.46 [8.06, 51.95]	1%	< 0.00001
Decreased platelet count	172/651	26.42%	86/719	11.96%	2.30 [1.48, 3.57]	61%	0.0002
Hyperbilirubinaemia	151/651	23.20%	84/719	11.68%	1.96 [1.16, 3.32]	70%	0.01
Pyrexia	140/651	21.51%	128/719	17.80%	1.19 [0.97, 1.47]	0%	0.10
Decreased appetite	102/485	21.03%	49/546	8.97%	2.31 [1.12, 4.77]	75%	0.02
Hypoalbuminaemia	103/497	20.72%	67/519	12.91%	1.61 [1.22, 2.13]	57%	0.0009
Diarrhoea	100/485	20.62%	47/546	8.61%	3.64 [0.66, 19.93]	93%	0.14
Grade 3–4 TRAEs							
Hypertension	79/485	16.29%	19/546	3.48%	4.29 [2.68, 6.86]	49%	< 0.00001
Increased aspartate aminotransferase	45/331	13.60%	26/346	7.51%	1.84 [1.18, 2.89]	49%	0.008
Decreased platelet count	41/485	8.45%	19/546	3.48%	2.28 [1.35, 3.84]	0%	0.002
Increased alanine aminotransferase	36/485	7.42%	26/546	4.76%	1.50 [0.93, 2.40]	0%	0.09
Diarrhoea	25/485	5.15%	0/546	0.00%	19.53 [3.71, 102.81]	0%	0.0005
Palmar-plantar erythrodysesthesia syndrome	16/331	4.83%	0/346	0.00%	17.87 [2.37, 134.62]	0%	0.005
Increased γ-glutamyltransferase	16/331	4.83%	2/346	0.58%	6.90 [1.85, 25.76]	0%	0.004
Proteinuria	18/485	3.71%	0/546	0.00%	14.63 [2.78, 76.97]	0%	0.002
Decreased neutrophil count	12/331	3.63%	4/346	1.16%	2.89 [1.00, 8.37]	0%	0.05
Hyperbilirubinaemia	16/485	3.30%	5/546	0.92%	3.21 [1.24, 8.31]	0%	0.02
Decreased white blood cell count	10/331	3.02%	1/346	0.29%	7.31 [1.33, 40.30]	0%	0.02
Hypokalaemia	10/331	3.02%	6/346	1.73%	1.74 [0.64, 4.72]	3%	0.28
Fatigue	13/485	2.68%	4/546	0.73%	3.58 [1.18, 10.92]	0%	0.02
Anemia	13/485	2.68%	5/546	0.92%	3.09 [1.11, 8.61]	0%	0.03
Post-embolisation syndrome	8/391	2.05%	13/443	2.93%	0.73 [0.31, 1.74]	0%	0.48

AVDs, Anti-VEGF drugs; CI, Confidence interval; I^2^, I-squared statistic; P, Probability; PD-1, Programmed cell death protein 1; PD-L1, Programmed death-ligand 1; PIs, PD-1/PD-L1 inhibitors; RR, Risk ratio; TACE, Transarterial chemoembolization; TPA, TACE plus PIs and AVDs; TRAEs, Treatment-related adverse events; VEGF, Vascular endothelial growth factor.

### Sensitivity analysis

Robustness checks of OS, PFS (per RECIST version 1.1), and total TRAEs were performed by sequentially omitting individual studies. The pooled outcomes were consistent, indicating that no individual study unduly influenced the results and that heterogeneity did not materially affect the overall effect estimates, supporting the stability of the findings (Supplementary Figure S7).

### Publication bias

**Figure 6:**
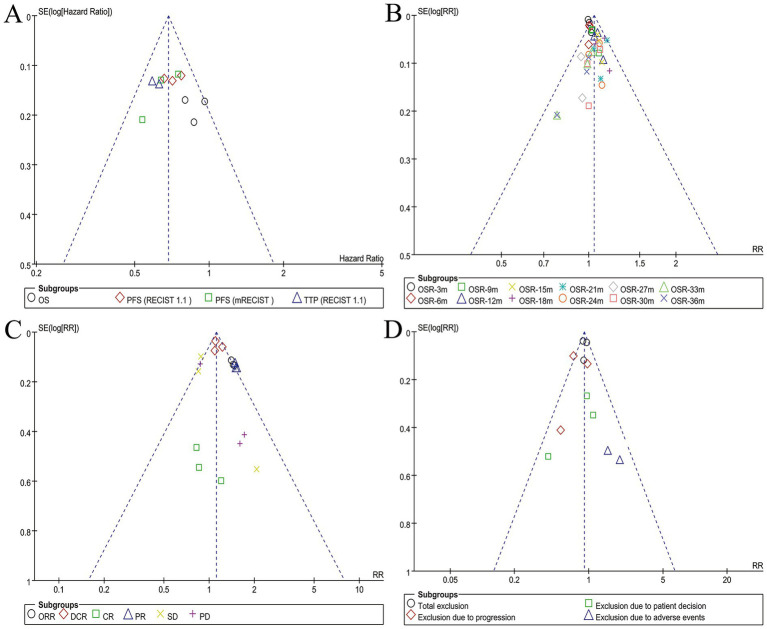
Evaluation of potential publication bias using funnel plots: **(A)** survival outcomes, **(B)** OSR, **(C)** tumor responses (RECIST version 1.1), and **(D)** exclusion at cutoff time.

Funnel plots of survival, OSR, responses (per RECIST version 1.1), and patient exclusions at the cutoff suggested a low likelihood of publication bias (Figure 6).

## Discussion

Combining systemic therapies with locoregional treatment is a major focus in UR/NM HCC research. Although TACE is the standard of care, its limited durability and its tendency to induce angiogenesis and immunosuppressive microenvironmental changes restrict long-term efficacy (17). In advanced disease, PIs plus AVDs have demonstrated OS benefit, providing a rationale for combining this strategy with TACE in earlier stages (18). Mechanistically, TACE-induced tumor necrosis may enhance antigen release, while anti-VEGF therapy may counteract post-embolization VEGF surges and improve immune cell infiltration, potentially facilitating immune checkpoint blockade (7). However, TPA increases treatment complexity, cost, and toxicity, raising concerns for patients with compromised liver function (19). In this meta-analysis of four RCTs, TPA significantly improved PFS, TTP, and ORR versus TACE alone, with consistent results across RECIST 1.1 and mRECIST criteria, and PFS benefits across most subgroups. However, Moderate heterogeneity was observed across trials, attributable to differences in PD-1/PD-L1 inhibitors, anti-VEGF agents, TACE techniques, and baseline characteristics. Nevertheless, no significant OS benefit was observed, and higher rates of severe and immune-related AEs were noted.

Survival remains the most clinically decisive endpoint when evaluating the value of intensified post-TACE therapy. In the present analysis, TPA achieved a statistically significant reduction in the risk of progression and tumor growth, as reflected by improved PFS and TTP, yet failed to demonstrate a statistically significant OS benefit, despite a numerically favorable trend. This dissociation between PFS and OS has also been observed in individual phase III trials, including EMERALD-1 and LEAP-012, in which marked PFS improvements were reported while OS data remained immature or nonsignificant at interim analyses (8, 9). Several factors may explain this phenomenon. First, OS in UR/NM HCC is heavily influenced by subsequent lines of therapy after progression, including crossover to immunotherapy, repeated TACE, or initiation of systemic treatments, which may dilute survival differences between early combination and sequential strategies (20). Second, intermediate-stage HCC is biologically heterogeneous, and long-term survival is strongly affected by baseline liver function, portal hypertension, and risk of hepatic decompensation, which may not be directly mitigated by improved tumor response alone (21). Third, increased toxicity in the TPA arm may lead to treatment interruptions, dose reductions, or hepatic injury, potentially offsetting gains achieved through tumor control (22). Notably, OS rate analyses in this study revealed a transient survival advantage favoring TPA at 12–18 months, suggesting that improved early disease control may translate into short-term survival benefit that becomes less apparent over longer follow-up due to competing risks and effective salvage therapies. This pattern supports the hypothesis that TPA primarily delays progression rather than fundamentally altering the natural history of the disease. Several trial-level differences may explain the moderate PFS heterogeneity. These include anti-VEGF type, TACE schedule, and baseline portal vein invasion. Despite this variability, all trials favored TPA (HR < 1.0), supporting the robustness of the pooled result (8, 9, 15, 16). Consequently, while PFS improvement is robust and clinically meaningful, its translation into durable survival benefit likely depends on both the depth and durability of immune-mediated tumor control as well as preservation of liver function over time (23). A critical point to emphasize is that a PFS benefit does not necessarily imply a survival benefit; therefore, the positive PFS result in this meta-analysis should not be interpreted as evidence of improved OS. Longer follow-up and mature OS data are therefore essential to determine whether early benefits ultimately translate into a survival advantage.

Tumor response provides an important surrogate marker of therapeutic activity and reflects the biological synergy between locoregional and systemic therapies. In this meta-analysis, TPA significantly improved ORR and DCR under both RECIST 1.1 and mRECIST criteria, with particularly notable gains in mRECIST-defined CR, which more accurately reflects viable tumor burden after locoregional therapy. This observation is clinically relevant, as mRECIST-based responses have been associated with improved survival following TACE in prior studies (24). The superior response profile of TPA supports the mechanistic hypothesis that anti-VEGF therapy normalizes tumor vasculature and reduces hypoxia-induced immunosuppression, thereby enhancing immune cell infiltration and drug delivery, while PD-1/PD-L1 blockade sustains cytotoxic T-cell activity triggered by TACE-induced antigen release (25). Clinically, improved response may facilitate downstaging, prolong TACE-free intervals, reduce the need for repeated embolization, and potentially preserve liver function in selected patients (26). However, the lack of corresponding OS improvement suggests that radiologic response alone may be insufficient to overcome long-term drivers of mortality, such as hepatic failure and systemic dissemination. Moreover, objective response does not guarantee eradication of microscopic residual disease, and durable immune surveillance may be required to prevent late recurrence (27). Therefore, although response improvement confirms biological activity and contributes to PFS benefit, it may not serve as a reliable surrogate for long-term survival in this population, particularly when effective post-progression therapies are widely available.

The safety profile of TPA is a critical determinant of its clinical applicability in patients with underlying chronic liver disease. This analysis demonstrates significantly higher rates of grade 3–4 or serious TEAEs, TRAEs, and irAEs in the TPA group. This toxicity burden is particularly relevant in HCC patients, who often have underlying cirrhosis and limited hepatic reserve, making them more vulnerable to treatment-related complications. The spectrum of increased toxicities reflects additive effects of PIs and AVDs, including hypertension, proteinuria, palmar–plantar erythrodysesthesia, hepatic enzyme elevation, hematologic abnormalities, and immune-mediated endocrine and gastrointestinal events (8, 9). Importantly, more patients in the TPA arm discontinued treatment due to AEs, potentially limiting sustained therapeutic exposure and undermining the intended synergistic effects of combination therapy (28). Such toxicity-related discontinuation may reduce treatment exposure and partly explain the lack of a significant OS benefit despite improved tumor control. In cirrhotic patients, even moderate hepatic injury may precipitate irreversible decompensation, which not only worsens quality of life but may also directly contribute to mortality. This raises the possibility that toxicity-related liver dysfunction may partially offset gains in tumor control, thereby contributing to the lack of significant OS benefit (29). From a clinical standpoint, hypertension and proteinuria require baseline blood pressure control and weekly monitoring. Hepatic enzyme elevations necessitate regular liver function tests, with dose interruption for severe cases. The three-fold higher discontinuation rate due to AEs (RR 3.12) likely explains why the PFS benefit did not translate into an OS advantage. Immune-related AEs and potential variceal bleeding risk should also be anticipated; pre-treatment endoscopy is advisable for patients with portal hypertension. The present results highlight the importance of meticulous patient selection, close monitoring, and early management of AEs. Patients with preserved liver function, low embolization burden, and good performance status may derive greater net benefit, whereas those with borderline hepatic reserve may be more vulnerable to treatment-related harm (30). Therefore, routine use of TPA for all patients after TACE may not be appropriate, and its application should be individualized, particularly in patients with preserved liver function and good performance status. Based on our subgroup findings, patients with Child-Pugh class A = 5 and non-HCV etiology appear more suitable for TPA, while those with Child-Pugh A = 6 or HCV-related HCC showed comparable PFS between arms and thus warrant more cautious consideration.

Several limitations of this analysis need to be acknowledged. First, although all included studies were high-quality RCTs, only four trials were available, and OS data in some trials remain immature, limiting definitive conclusions regarding long-term survival. In addition, data from CARES-005 and TALENTACE were derived from conference abstracts rather than fully published articles, which introduces potential bias due to incomplete reporting and lack of full peer review, potentially affecting the completeness and reliability of outcome reporting. Second, clinical heterogeneity exists across trials in terms of PIs, AVDs, TACE techniques, treatment schedules, and post-progression therapies, which warrants caution when generalizing our findings. This heterogeneity contributed to variability in effect sizes, but sensitivity analyses confirmed the main conclusions remained stable. Third, this evaluation utilized aggregated study-level rather than patient-level data, precluding more refined assessments of dynamic liver function changes, detailed treatment sequencing, and interactions between baseline characteristics and treatment effects. Fourth, subgroup analyses, although largely consistent with overall PFS benefit, may be underpowered to detect meaningful differences in specific populations. Fifth, finer BCLC subcategories (e.g., B1, B2) and tumor differentiation could not be examined due to lack of uniform reporting across trials, which warrants future investigation. Finally, trial populations were highly selected, which may limit generalizability to real-world settings where comorbidities and treatment adherence vary substantially. These limitations highlight the importance of longer follow-up, real-world studies, and individual patient data meta-analyses to better define which patients are most likely to derive meaningful benefit from TPA.

## Conclusion

In patients with UR/NM HCC, the addition of PIs plus AVDs to TACE significantly improves disease control, as reflected by enhanced PFS, TTP, and tumor response rates, but does not yet confer a statistically significant OS benefit. Subgroup analyses indicated that the PFS advantage of the TPA group was consistent across nearly all subgroups. This regimen is associated with increased severe AEs, which should be carefully balanced against its benefits, especially in patients with compromised liver function. Consequently, the decision to employ this strategy should be highly individualized, carefully balancing the potential for delayed disease progression against the risks of heightened toxicity, particularly in patients with compromised liver function.

## Data Availability

The datasets presented in this study can be found in online repositories. The names of the repository/repositories and accession number(s) can be found in the article/Supplementary material.

## Supplementary material

SUPPLEMENTARY FIGURE S1 Assessment of study-level risk of bias using the Cochrane framework.

SUPPLEMENTARY FIGURE S2 Forest plots illustrating progression-free survival rates (PFSR) for TPA versus TACE across follow-up intervals of 3–36 months.

SUPPLEMENTARY FIGURE S3 Forest plots illustrating overall survival rates (OSR) for TPA versus TACE across follow-up intervals of 3–36 months.

SUPPLEMENTARY FIGURE S4 Forest plots illustrating duration of response for TPA versus TACE.

SUPPLEMENTARY FIGURE S5 Forest plots illustrating tumor response rates according to mRECIST for TPA versus TACE.

SUPPLEMENTARY FIGURE S6 Forest plots summarizing adverse events for TPA versus TACE.

SUPPLEMENTARY FIGURE S7 Sensitivity analyses for OS **(A)**, PFS (RECIST version 1.1) **(B)**, and total TRAEs **(C)**.

## Glossary

AEs: Adverse events
ALT: Alanine aminotransferase
AST: Aspartate aminotransferase
AVDs: Anti-VEGF drugs
BCLC: Barcelona Clinic Liver Cancer
CI: Confidence interval
CR: Complete response
DCR: Disease control rate
ECOG PS: Eastern Cooperative Oncology Group Performance Status
GGT: Gamma-glutamyl transferase
GRADE: Grading of Recommendations Assessment, Development and Evaluation
HCC: Hepatocellular carcinoma
HR: Hazard ratio
*I^2^*: I-squared statistic
ICIs: Immune checkpoint inhibitors
irAEs: Immune-related adverse events
MD: Mean difference
M/F: Male/Female
mRECIST: Modified Response Evaluation Criteria in Solid Tumors
NM: Non-metastatic
ORR: Objective response rate
OS: Overall survival
OSR: Overall survival rate
P: Probability
PD: Progressive disease
PD-1: Programmed cell death protein 1
PD-L1: Programmed death-ligand 1
PFS: Progression-free survival
PFSR: Progression-free survival rate
PIs: PD-1/PD-L1 inhibitors
PR: Partial response
PRISMA: Preferred reporting items for systematic reviews and meta-analyses
PROSPERO: International prospective register of systematic reviews
RCT: Randomized controlled trial
RECIST: Response Evaluation Criteria in Solid Tumors
RR: Risk ratio
SD: Stable disease
TACE: Transarterial chemoembolisation
TEAEs: Treatment-emergent adverse events
TPA: TACE plus PIs and AVDs
TRAEs: Treatment-related adverse events
TTP: Time to progression
UR: Unresectable
VEGF: Vascular endothelial growth factor.
