# Supplementary figures and images for: The benefit and risk of adding PD-1/PD-L1 inhibitors plus anti-VEGF drugs to transarterial chemoembolisation for unresectable, non-metastatic hepatocellular carcinoma: a pooled analysis of four RCTs
Source: Front Med (Lausanne). 2026 May 25;13:1792746. doi: 10.3389/fmed.2026.1792746 (PMC13244568; doi:10.3389/fmed.2026.1792746)

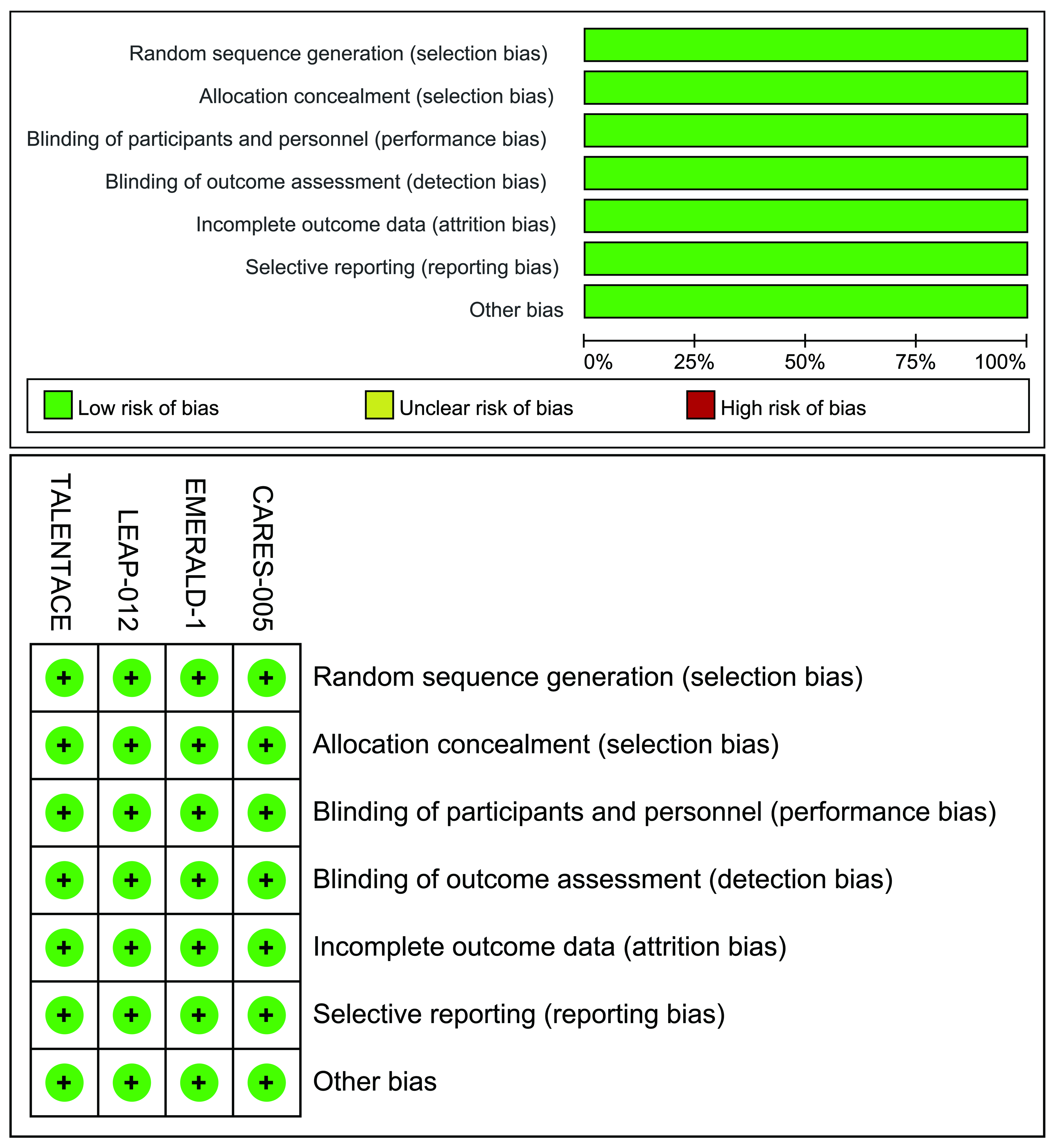

Supplement: SUPPLEMENTARY FIGURE S1 — Assessment of study-level risk of bias using the Cochrane framework. [file Image_1.tif]

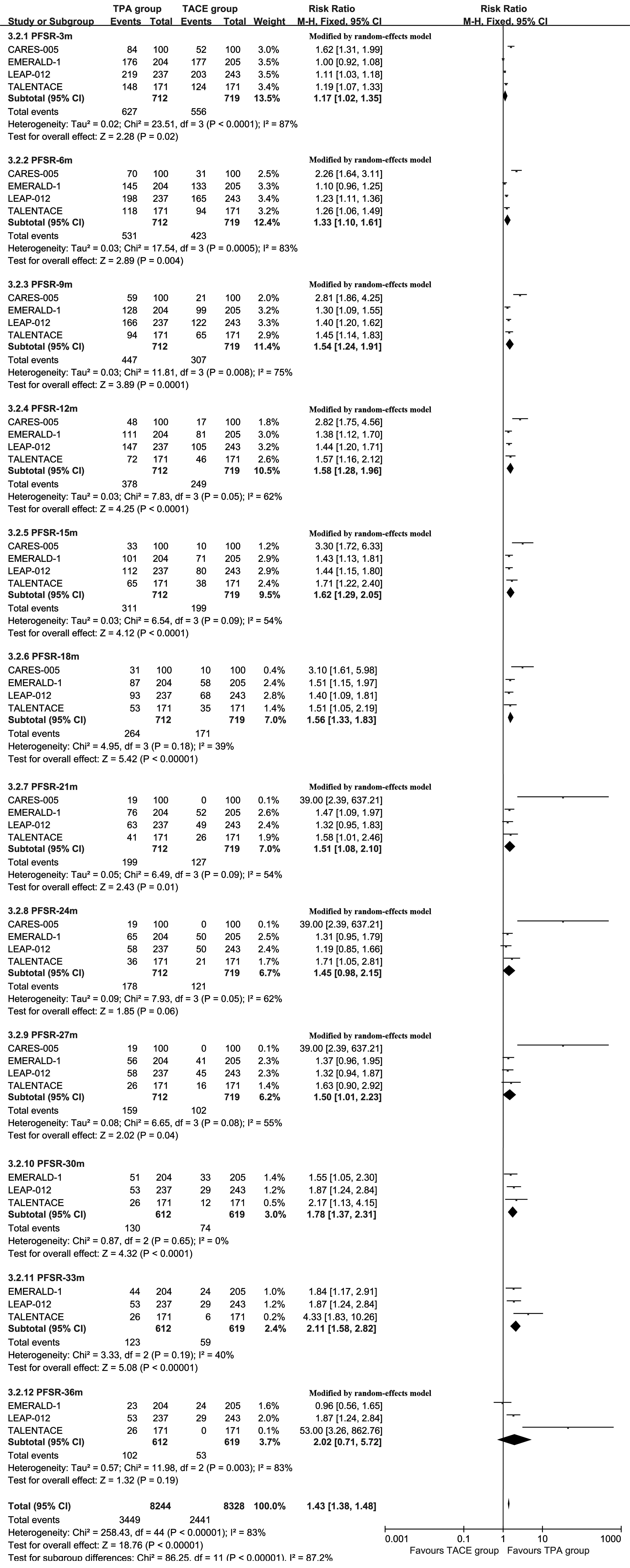

Supplement: SUPPLEMENTARY FIGURE S2 — Forest plots illustrating progression-free survival rates (PFSR) for TPA versus TACE across follow-up intervals of 3–36 months. [file Image_2.tif]

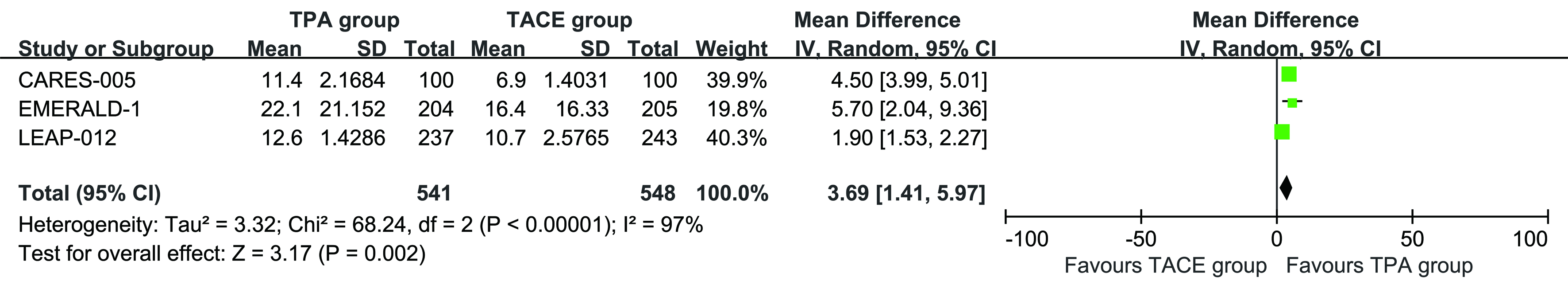

Supplement: SUPPLEMENTARY FIGURE S4 — Forest plots illustrating duration of response for TPA versus TACE. [file Image_4.tif]

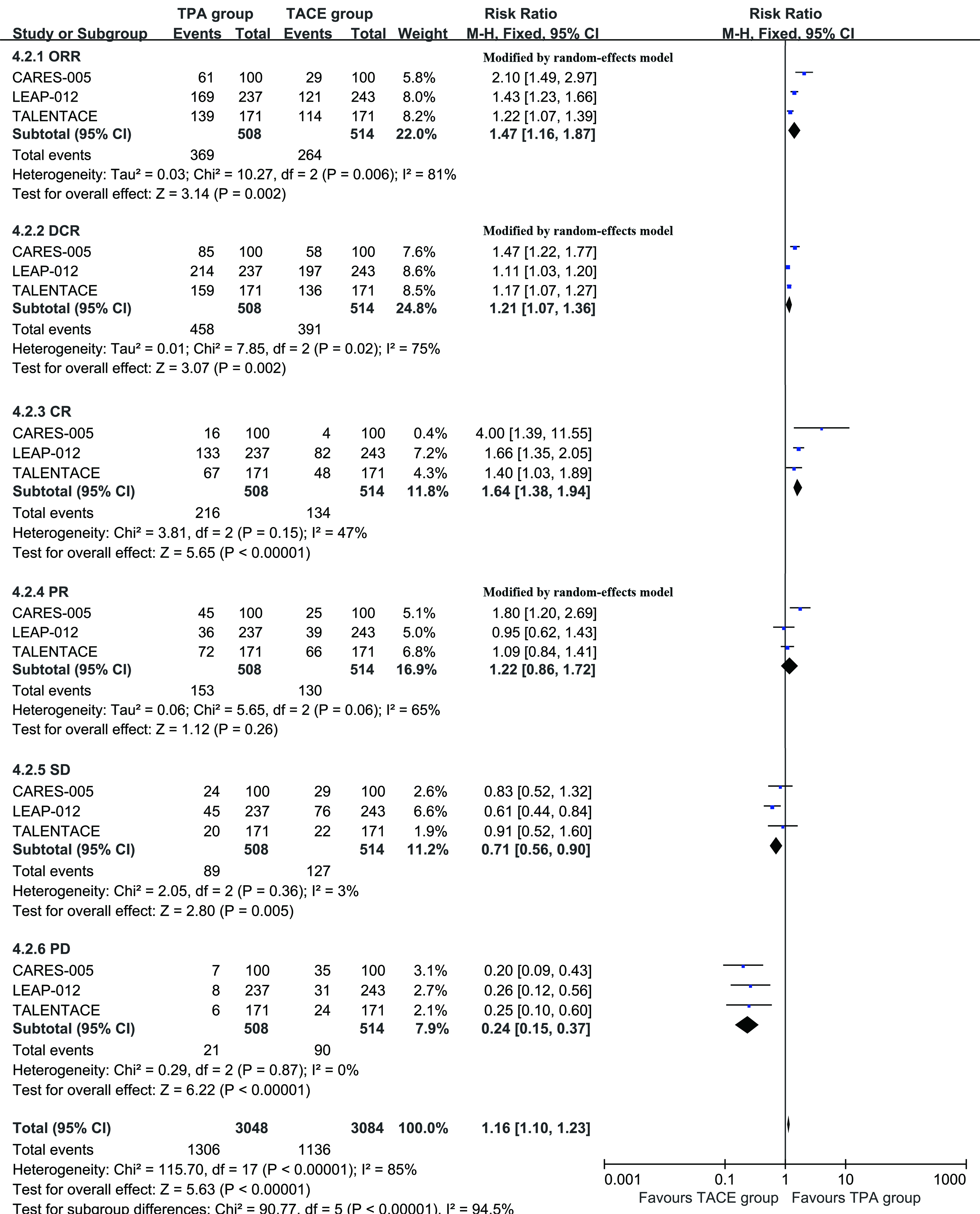

Supplement: SUPPLEMENTARY FIGURE S5 — Forest plots illustrating tumor response rates according to mRECIST for TPA versus TACE. [file Image_5.tif]

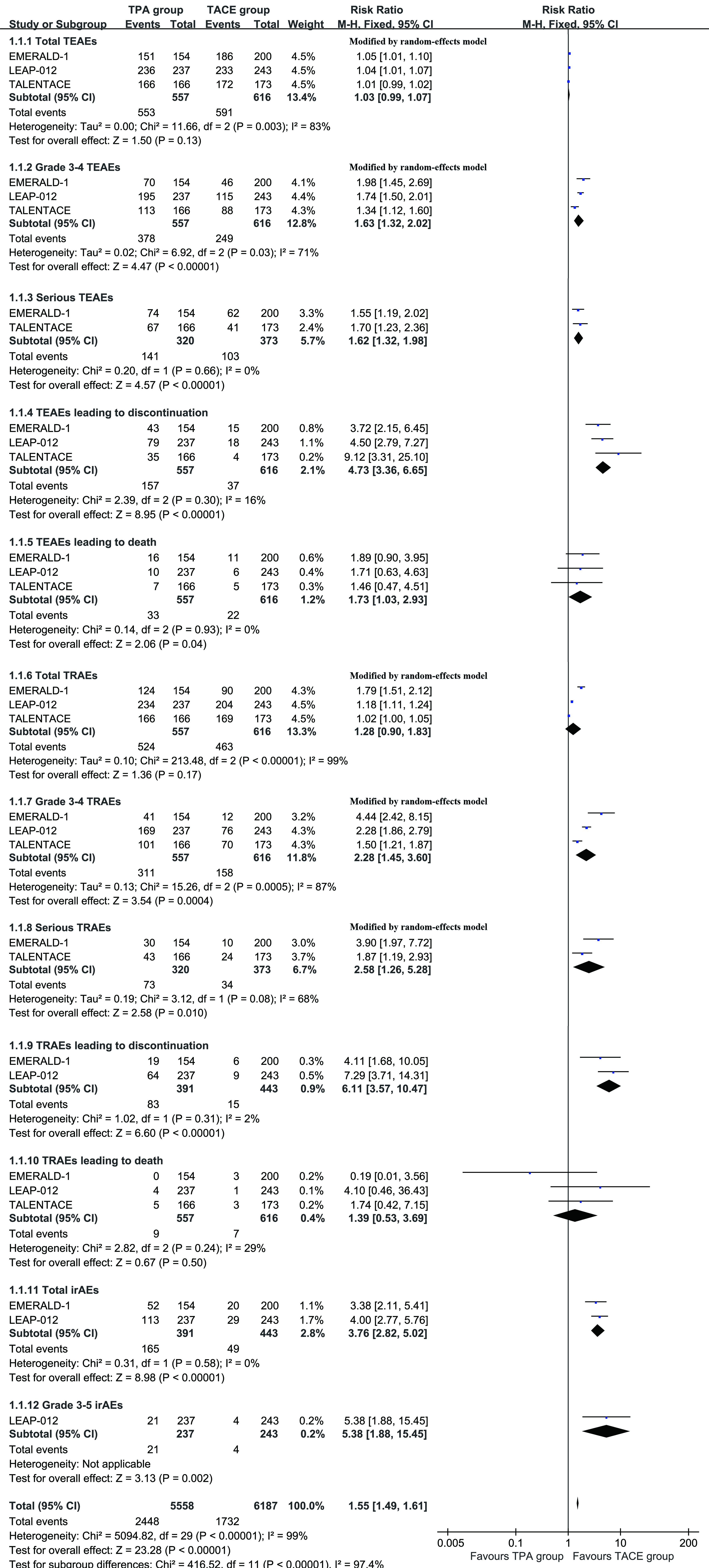

Supplement: SUPPLEMENTARY FIGURE S6 — Forest plots summarizing adverse events for TPA versus TACE. [file Image_6.tif]

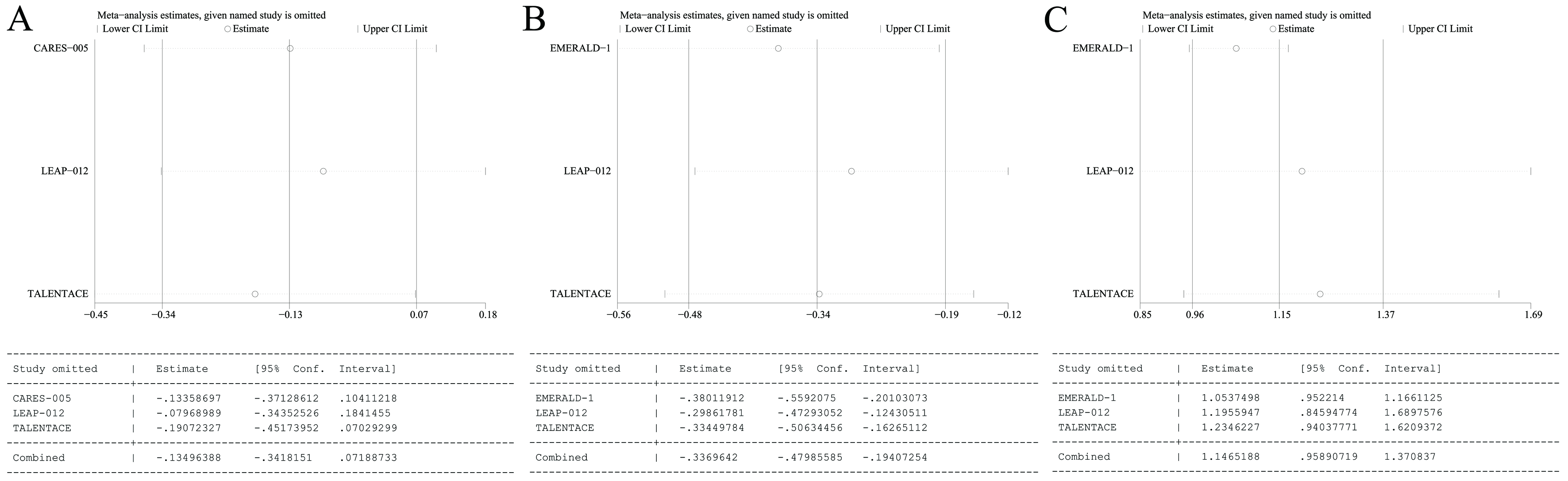

Supplement: SUPPLEMENTARY FIGURE S7 — Sensitivity analyses for OS (A), PFS (RECIST version 1.1) (B), and total TRAEs (C). [file Image_7.tif]
