# Supplementary material for: The benefit and risk of adding PD-1/PD-L1 inhibitors plus anti-VEGF drugs to transarterial chemoembolisation for unresectable, non-metastatic hepatocellular carcinoma: a pooled analysis of four RCTs
Source: Front Med (Lausanne). 2026 May 25;13:1792746. doi: 10.3389/fmed.2026.1792746 (PMC13244568; doi:10.3389/fmed.2026.1792746)
Supplement: Supplementary file 8 [file Table_1.docx]

**Table S1** Comprehensive search strategies.

| **PubMed**  The database was searched on December 27, 2025, n=77.  Search Strategy:  #1 Search: Transarterial chemoembolization[Title/Abstract] OR Chemoembolization[Title/Abstract] OR TACE[Title/Abstract] OR Transarterial Chemo-embolization[Title/Abstract] OR Intra-arterial Chemoembolization[Title/Abstract] OR Arterial Chemoembolization[Title/Abstract] OR Chemoembolization[Title/Abstract] OR Chemo-embolization[Title/Abstract] OR Chemoembolisation[Title/Abstract] OR Chemo-embolisation[Title/Abstract] Sort by: Most Recent n = 14,379  #2 Search: Immunotherapy[Title/Abstract] OR Immune checkpoint inhibitors[Title/Abstract] OR ICIs[Title/Abstract] OR PD-1 inhibitor[Title/Abstract] OR PD-L1 inhibitor[Title/Abstract] OR PD-1/PD-L1 inhibitor[Title/Abstract] OR Nivolumab[Title/Abstract] OR Pembrolizumab[Title/Abstract] OR Toripalimab[Title/Abstract] OR Sintilimab[Title/Abstract] OR Camrelizumab[Title/Abstract] OR Tislelizumab[Title/Abstract] OR Penpulimab[Title/Abstract] OR Zimberelimab[Title/Abstract] OR Serplulimab[Title/Abstract] OR Durvalumab[Title/Abstract] OR Atezolizumab[Title/Abstract] OR Envafolimab[Title/Abstract] OR Sugemalimab[Title/Abstract] OR Adebrelimab[Title/Abstract] OR Cemiplimab[Title/Abstract] OR Retifanlimab[Title/Abstract] OR Envafolimab[Title/Abstract] OR Cadonilimab[Title/Abstract] OR Dostarlimab[Title/Abstract] OR Socazolimab[Title/Abstract] OR Avelumab[Title/Abstract] OR Cosibelimab[Title/Abstract] OR Balstilimab[Title/Abstract] OR Sasanlimab[Title/Abstract] OR Budigalimab[Title/Abstract] OR Ivonescimab[Title/Abstract] OR Spartalizumab[Title/Abstract] OR Cetrelimab[Title/Abstract] Sort by: Most Recent n = 207,791  #3 Search: Carcinomas, Hepatocellular[Title/Abstract] OR Hepatocellular Carcinomas[Title/Abstract] OR Hepatocellular Carcinoma[Title/Abstract] OR Hepatoma[Title/Abstract] OR Hepatomas[Title/Abstract] OR Liver Cancer[Title/Abstract] OR Adult Liver Cancer[Title/Abstract] OR Adult Liver Cancers[Title/Abstract] OR Cancer, Adult Liver[Title/Abstract] OR Cancers, Adult Liver[Title/Abstract] OR Liver Cancers[Title/Abstract] OR Liver Cell Carcinoma[Title/Abstract] OR Carcinoma, Liver Cell[Title/Abstract] OR Carcinomas, Liver Cell[Title/Abstract] OR Cell Carcinoma, Liver[Title/Abstract] OR Cell Carcinomas, Liver[Title/Abstract] OR Liver Cell Carcinomas[Title/Abstract] OR Liver Cell Carcinoma[Title/Abstract] Sort by: Most Recent n = 191,488  #4 Randomized[Title/Abstract]) OR Randomly[Title/Abstract] OR Randomised[Title/Abstract] Sort by: Most Recent n = 1,307,665  #1 and #2 and #3 and #4 n = 77 |
| --- |
| **Web of Science**  The database was searched on December 27, 2025, n=77.  Search Strategy:  Transarterial chemoembolization OR Chemoembolization OR TACE OR Transarterial Chemo-embolization OR Intra-arterial Chemoembolization OR Arterial Chemoembolization OR Chemoembolization OR Chemo-embolization OR Chemoembolisation OR Chemo-embolisation (Abstract) and Immunotherapy OR Immune checkpoint inhibitors OR ICIs OR PD-1 inhibitor OR PD-L1 inhibitor OR PD-1/PD-L1 inhibitor OR Nivolumab OR Pembrolizumab OR Toripalimab OR Sintilimab OR Camrelizumab OR Tislelizumab OR Penpulimab OR Zimberelimab OR Serplulimab OR Durvalumab OR Atezolizumab OR Envafolimab OR Sugemalimab OR Adebrelimab OR Cemiplimab OR Retifanlimab OR Envafolimab OR Cadonilimab OR Dostarlimab OR Socazolimab OR Avelumab OR Cosibelimab OR Balstilimab OR Sasanlimab OR Budigalimab OR Ivonescimab OR Spartalizumab OR Cetrelimab (Abstract) and Carcinomas, Hepatocellular OR Hepatocellular Carcinomas OR Hepatocellular Carcinoma OR Hepatoma OR Hepatomas OR Liver Cancer OR Adult Liver Cancer OR Adult Liver Cancers OR Cancer, Adult Liver OR Cancers, Adult Liver OR Liver Cancers OR Liver Cell Carcinoma OR Carcinoma, Liver Cell OR Carcinomas, Liver Cell OR Cell Carcinoma, Liver OR Cell Carcinomas, Liver OR Liver Cell Carcinomas OR Liver Cell Carcinoma (Abstract) and Randomized OR Randomly OR Randomised (Abstract) |
| **EMBASE**  The database was searched on December 27, 2025, n=214.  Search Strategy:  (Transarterial chemoembolization:ti,ab,kw OR Chemoembolization:ti,ab,kw OR TACE:ti,ab,kw OR Transarterial Chemo-embolization:ti,ab,kw OR Intra-arterial Chemoembolization:ti,ab,kw OR Arterial Chemoembolization:ti,ab,kw OR Chemoembolization:ti,ab,kw OR Chemo-embolization:ti,ab,kw OR Chemoembolisation:ti,ab,kw OR Chemo-embolisation:ti,ab,kw) AND (Immunotherapy:ti,ab,kw OR Immune checkpoint inhibitors:ti,ab,kw OR ICIs:ti,ab,kw OR PD-1 inhibitor:ti,ab,kw OR PD-L1 inhibitor:ti,ab,kw OR PD-1/PD-L1 inhibitor:ti,ab,kw OR Nivolumab:ti,ab,kw OR Pembrolizumab:ti,ab,kw OR Toripalimab:ti,ab,kw OR Sintilimab:ti,ab,kw OR Camrelizumab:ti,ab,kw OR Tislelizumab:ti,ab,kw OR Penpulimab:ti,ab,kw OR Zimberelimab:ti,ab,kw OR Serplulimab:ti,ab,kw OR Durvalumab:ti,ab,kw OR Atezolizumab:ti,ab,kw OR Envafolimab:ti,ab,kw OR Sugemalimab:ti,ab,kw OR Adebrelimab:ti,ab,kw OR Cemiplimab:ti,ab,kw OR Retifanlimab:ti,ab,kw OR Envafolimab:ti,ab,kw OR Cadonilimab:ti,ab,kw OR Dostarlimab:ti,ab,kw OR Socazolimab:ti,ab,kw OR Avelumab:ti,ab,kw OR Cosibelimab:ti,ab,kw OR Balstilimab:ti,ab,kw OR Sasanlimab:ti,ab,kw OR Budigalimab:ti,ab,kw OR Ivonescimab:ti,ab,kw OR Spartalizumab:ti,ab,kw OR Cetrelimab:ti,ab,kw) AND (Carcinomas, Hepatocellular:ti,ab,kw OR Hepatocellular Carcinomas:ti,ab,kw OR Hepatocellular Carcinoma:ti,ab,kw OR Hepatoma:ti,ab,kw OR Hepatomas:ti,ab,kw OR Liver Cancer:ti,ab,kw OR Adult Liver Cancer:ti,ab,kw OR Adult Liver Cancers:ti,ab,kw OR Cancer, Adult Liver:ti,ab,kw OR Cancers, Adult Liver:ti,ab,kw OR Liver Cancers:ti,ab,kw OR Liver Cell Carcinoma:ti,ab,kw OR Carcinoma, Liver Cell:ti,ab,kw OR Carcinomas, Liver Cell:ti,ab,kw OR Cell Carcinoma, Liver:ti,ab,kw OR Cell Carcinomas, Liver:ti,ab,kw OR Liver Cell Carcinomas:ti,ab,kw OR Liver Cell Carcinoma:ti,ab,kw) AND **(Randomly**:ti,ab,kw **OR Randomised**:ti,ab,kw **OR Randomized**:ti,ab,kw**)** |
| **Cochrane Library**  The database was searched on December 27, 2025, n=183.  Search Strategy:  Transarterial chemoembolization OR Chemoembolization OR TACE OR Transarterial Chemo-embolization OR Intra-arterial Chemoembolization OR Arterial Chemoembolization OR Chemoembolization OR Chemo-embolization OR Chemoembolisation OR Chemo-embolisation in Title Abstract Keyword AND Immunotherapy OR Immune checkpoint inhibitors OR ICIs OR PD-1 inhibitor OR PD-L1 inhibitor OR PD-1/PD-L1 inhibitor OR Nivolumab OR Pembrolizumab OR Toripalimab OR Sintilimab OR Camrelizumab OR Tislelizumab OR Penpulimab OR Zimberelimab OR Serplulimab OR Durvalumab OR Atezolizumab OR Envafolimab OR Sugemalimab OR Adebrelimab OR Cemiplimab OR Retifanlimab OR Envafolimab OR Cadonilimab OR Dostarlimab OR Socazolimab OR Avelumab OR Cosibelimab OR Balstilimab OR Sasanlimab OR Budigalimab OR Ivonescimab OR Spartalizumab OR Cetrelimab in Title Abstract Keyword AND Carcinomas, Hepatocellular OR Hepatocellular Carcinomas OR Hepatocellular Carcinoma OR Hepatoma OR Hepatomas OR Liver Cancer OR Adult Liver Cancer OR Adult Liver Cancers OR Cancer, Adult Liver OR Cancers, Adult Liver OR Liver Cancers OR Liver Cell Carcinoma OR Carcinoma, Liver Cell OR Carcinomas, Liver Cell OR Cell Carcinoma, Liver OR Cell Carcinomas, Liver OR Liver Cell Carcinomas OR Liver Cell Carcinoma in Title Abstract Keyword AND Randomized OR Randomly OR Randomised in Title Abstract Keyword - (Word variations have been searched) |
| **ScienceDirect**  The database was searched on December 27, 2025, n=181.  Search Strategy:  Title, abstract, keywords: ((“Transarterial chemoembolization” OR “Chemoembolization” OR “TACE” OR “Transarterial” Chemo-embolization” OR “Intra-arterial Chemoembolization” OR “Arterial Chemoembolization” OR “Chemoembolization” OR “Chemo-embolization” OR “Chemoembolisation” OR “Chemo-embolisation”) AND (“Immunotherapy” OR “Immune checkpoint inhibitors” OR “ICIs” OR “PD-1 inhibitor” OR “PD-L1 inhibitor” OR “PD-1/PD-L1 inhibitor” OR “Nivolumab” OR “Pembrolizumab” OR “Toripalimab” OR “Sintilimab” OR “Camrelizumab” OR “Tislelizumab” OR “Penpulimab” OR “Zimberelimab” OR “Serplulimab” OR “Durvalumab” OR “Atezolizumab” OR “Envafolimab” OR “Sugemalimab” OR “Adebrelimab” OR “Cemiplimab” OR “Retifanlimab” OR “Envafolimab” OR “Cadonilimab” OR “Dostarlimab” OR “Socazolimab” OR “Avelumab” OR “Cosibelimab” OR “Balstilimab” OR “Sasanlimab” OR “Budigalimab” OR “Ivonescimab” OR “Spartalizumab” OR “Cetrelimab”) AND (“Carcinomas, Hepatocellular” OR “Hepatocellular Carcinomas” OR “Hepatocellular Carcinoma” OR “Hepatoma” OR “Hepatomas” OR “Liver Cancer” OR “Adult Liver Cancer” OR “Adult Liver Cancers” OR “Cancer, Adult Liver” OR “Cancers, Adult Liver” OR “Liver Cancers” OR “Liver Cell Carcinoma” OR “Carcinoma, Liver Cell” OR “Carcinomas, Liver Cell” OR “Cell Carcinoma, Liver” OR “Cell Carcinomas, Liver” OR “Liver Cell Carcinomas” OR “Liver Cell Carcinoma”) AND (“**Randomized**” **OR Randomly**” **OR** “**Randomised**”)) |
| **Scopus**  The database was searched on December 27, 2025, n=27.  Search Strategy:  (TITLE-ABS-KEY (Transarterial chemoembolization OR Chemoembolization OR TACE OR Transarterial Chemo-embolization OR Intra-arterial Chemoembolization OR Arterial Chemoembolization OR Chemoembolization OR Chemo-embolization OR Chemoembolisation OR Chemo-embolisation) AND TITLE-ABS-KEY (Immunotherapy OR Immune checkpoint inhibitors OR ICIs OR PD-1 inhibitor OR PD-L1 inhibitor OR PD-1/PD-L1 inhibitor OR Nivolumab OR Pembrolizumab OR Toripalimab OR Sintilimab OR Camrelizumab OR Tislelizumab OR Penpulimab OR Zimberelimab OR Serplulimab OR Durvalumab OR Atezolizumab OR Envafolimab OR Sugemalimab OR Adebrelimab OR Cemiplimab OR Retifanlimab OR Envafolimab OR Cadonilimab OR Dostarlimab OR Socazolimab OR Avelumab OR Cosibelimab OR Balstilimab OR Sasanlimab OR Budigalimab OR Ivonescimab OR Spartalizumab OR Cetrelimab) AND TITLE-ABS-KEY (Carcinomas, Hepatocellular OR Hepatocellular Carcinomas OR Hepatocellular Carcinoma OR Hepatoma OR Hepatomas OR Liver Cancer OR Adult Liver Cancer OR Adult Liver Cancers OR Cancer, Adult Liver OR Cancers, Adult Liver OR Liver Cancers OR Liver Cell Carcinoma OR Carcinoma, Liver Cell OR Carcinomas, Liver Cell OR Cell Carcinoma, Liver OR Cell Carcinomas, Liver OR Liver Cell Carcinomas OR Liver Cell Carcinoma) AND TITLE-ABS-KEY (Randomized OR Randomly OR Randomised)) |

**Note:** The combined text and medical subject heading (MeSH) terms used were: “**Transarterial chemoembolization**”, “**PD-1/PD-L1 inhibitors**”, “Hepatocellular carcinoma”, and “**Randomized**”.
