# Supplementary material for: The benefit and risk of adding PD-1/PD-L1 inhibitors plus anti-VEGF drugs to transarterial chemoembolisation for unresectable, non-metastatic hepatocellular carcinoma: a pooled analysis of four RCTs
Source: Front Med (Lausanne). 2026 May 25;13:1792746. doi: 10.3389/fmed.2026.1792746 (PMC13244568; doi:10.3389/fmed.2026.1792746)
Supplement: Supplementary file 9 [file Table_2.doc]

**Table S2** Quality appraisal of included trials using the Jadad scoring system.

| **Study** | | **Randomization** | **Concealment of allocation** | **Double blinding** | **Withdrawals and dropouts** | **Quality (score)** |
| --- | --- | --- | --- | --- | --- | --- |
| CARES-005 [15] | NCT04559607 | ** | ** | * | * | 7 |
| EMERALD-1 [8] | NCT03778957 | ** | ** | * | * | 7 |
| LEAP-012 [9] | NCT04246177 | ** | ** | * | * | 7 |
| TALENTACE [16] | NCT04712643 | ** | ** | * | * | 7 |
