# Supplementary material for: The benefit and risk of adding PD-1/PD-L1 inhibitors plus anti-VEGF drugs to transarterial chemoembolisation for unresectable, non-metastatic hepatocellular carcinoma: a pooled analysis of four RCTs
Source: Front Med (Lausanne). 2026 May 25;13:1792746. doi: 10.3389/fmed.2026.1792746 (PMC13244568; doi:10.3389/fmed.2026.1792746)
Supplement: Supplementary file 10 [file Table_3.doc]

**Table S3** Grading of evidence certainty according to GRADE methodology stratified by intervention and study design.

| **Primary outcomes** | **No. of Participants** | | **Differences (95%CI) ^a^** | **Quality Assessment** | | | | | **Quality** |
| --- | --- | --- | --- | --- | --- | --- | --- | --- | --- |
|  | **TPA** | **TACE** |  | **Risk of Bias ^b^** | **Inconsistency** | **Indirectness** | **Imprecision** | **Publication Bias ^c^** |  |
| **Survival** |  |  |  |  |  |  |  |  |  |
| OS | 508 | 514 | 0.87 [0.71, 1.07] | Low | No inconsistency | No indirectness | No imprecision | Unlikely | High |
| PFS (RECIST 1.1) | 712 | 719 | 0.62 [0.47, 0.81] | Low | Serious (-1) | No indirectness | No imprecision | Unlikely | Medium |
| PFS (mRECIST) | 541 | 548 | 0.56 [0.37, 0.84] | Low | Serious (-1) | No indirectness | No imprecision | Unlikely | Medium |
| TTP (RECIST 1.1) | 441 | 448 | 0.61 [0.51, 0.73] | Low | No inconsistency | No indirectness | No imprecision | Unlikely | High |
| **Survival rate** |  |  |  |  |  |  |  |  |  |
| **PFSR (RECIST 1.1)** |  |  |  |  |  |  |  |  |  |
| PFSR-3m | 627/712 | 556/719 | 1.17 [1.02, 1.35] | Low | Serious (-1) | No indirectness | No imprecision | Unlikely | Medium |
| PFSR-6m | 531/712 | 423/719 | 1.33 [1.10, 1.61] | Low | Serious (-1) | No indirectness | No imprecision | Unlikely | Medium |
| PFSR-9m | 447/712 | 307/719 | 1.54 [1.24, 1.91] | Low | Serious (-1) | No indirectness | No imprecision | Unlikely | Medium |
| PFSR-12m | 378/712 | 249/719 | 1.58 [1.28, 1.96] | Low | Serious (-1) | No indirectness | No imprecision | Unlikely | Medium |
| PFSR-15m | 311/712 | 199/719 | 1.62 [1.29, 2.05] | Low | Serious (-1) | No indirectness | No imprecision | Unlikely | Medium |
| PFSR-18m | 264/712 | 171/719 | 1.56 [1.33, 1.83] | Low | No inconsistency | No indirectness | No imprecision | Unlikely | High |
| PFSR-21m | 199/712 | 127/719 | 1.51 [1.08, 2.10] | Low | Serious (-1) | No indirectness | No imprecision | Unlikely | Medium |
| PFSR-24m | 178/712 | 121/719 | 1.45 [0.98, 2.15] | Low | Serious (-1) | No indirectness | No imprecision | Unlikely | Medium |
| PFSR-27m | 159/712 | 102/719 | 1.50 [1.01, 2.23] | Low | Serious (-1) | No indirectness | No imprecision | Unlikely | Medium |
| PFSR-30m | 130/612 | 74/619 | 1.78 [1.37, 2.31] | Low | No inconsistency | No indirectness | No imprecision | Unlikely | High |
| PFSR-33m | 123/612 | 59/619 | 2.11 [1.58, 2.82] | Low | No inconsistency | No indirectness | No imprecision | Unlikely | High |
| PFSR-36m | 102/612 | 53/619 | 2.02 [0.71, 5.72] | Low | Serious (-1) | No indirectness | No imprecision | Unlikely | Medium |
| **OSR** | | | |  |  |  |  |  |  |
| OSR-3m | 497/508 | 501/514 | 1.00 [0.99, 1.02] | Low | No inconsistency | No indirectness | No imprecision | Unlikely | High |
| OSR-6m | 473/508 | 476/514 | 1.01 [0.97, 1.04] | Low | No inconsistency | No indirectness | No imprecision | Unlikely | High |
| OSR-9m | 454/508 | 443/514 | 1.04 [0.99, 1.09] | Low | No inconsistency | No indirectness | No imprecision | Unlikely | High |
| OSR-12m | 436/508 | 412/514 | 1.07 [1.01, 1.13] | Low | No inconsistency | No indirectness | No imprecision | Unlikely | High |
| OSR-15m | 418/508 | 391/514 | 1.08 [1.02, 1.15] | Low | No inconsistency | No indirectness | No imprecision | Unlikely | High |
| OSR-18m | 397/508 | 361/514 | 1.11 [1.04, 1.20] | Low | No inconsistency | No indirectness | No imprecision | Unlikely | High |
| OSR-21m | 371/508 | 339/514 | 1.11 [1.02, 1.20] | Low | No inconsistency | No indirectness | No imprecision | Unlikely | High |
| OSR-24m | 335/508 | 320/514 | 1.06 [0.97, 1.16] | Low | No inconsistency | No indirectness | No imprecision | Unlikely | High |
| OSR-27m | 305/508 | 304/514 | 1.02 [0.92, 1.12] | Low | No inconsistency | No indirectness | No imprecision | Unlikely | High |
| OSR-30m | 279/508 | 270/514 | 1.05 [0.94, 1.17] | Low | No inconsistency | No indirectness | No imprecision | Unlikely | High |
| OSR-33m | 258/508 | 267/514 | 0.98 [0.87, 1.10] | Low | No inconsistency | No indirectness | No imprecision | Unlikely | High |
| OSR-36m | 228/508 | 240/514 | 0.96 [0.84, 1.10] | Low | No inconsistency | No indirectness | No imprecision | Unlikely | High |
| **Subgroup analysis of PFS (RECIST 1.1)** |  |  |  |  |  |  |  |  |  |
| All patients | 1431 | | 0.62 [0.47, 0.81] | Low | Serious (-1) | No indirectness | No imprecision | Unlikely | Medium |
| Age < 65 years | 765 | | 0.61 [0.44, 0.85] | Low | Serious (-1) | No indirectness | No imprecision | Unlikely | Medium |
| Age > 65 years | 666 | | 0.67 [0.55, 0.81] | Low | No inconsistency | No indirectness | No imprecision | Unlikely | High |
| Sex - Female | 258 | | 0.78 [0.57, 1.05] | Low | No inconsistency | No indirectness | No imprecision | Unlikely | High |
| Sex - Male | 1173 | | 0.59 [0.44, 0.80] | Low | Serious (-1) | No indirectness | No imprecision | Unlikely | Medium |
| ECOG PS - 0 | 1173 | | 0.65 [0.57, 0.76] | Low | No inconsistency | No indirectness | No imprecision | Unlikely | High |
| ECOG PS - 1 | 257 | | 0.63 [0.33, 1.23] | Low | Serious (-1) | No indirectness | No imprecision | Unlikely | Medium |
| Geographic region - Japan | 64 | | 0.78 [0.43, 1.40] | Low | No inconsistency | No indirectness | No imprecision | Unlikely | High |
| Geographic region - Asia (not including Japan) | 994 | | 0.59 [0.43, 0.80] | Low | Serious (-1) | No indirectness | No imprecision | Unlikely | Medium |
| Aetiology of liver disease - Hepatitis B | 883 | | 0.61 [0.46, 0.81] | Low | Serious (-1) | No indirectness | No imprecision | Unlikely | Medium |
| Aetiology of liver disease - Hepatitis C | 190 | | 0.84 [0.59, 1.20] | Low | No inconsistency | No indirectness | No imprecision | Unlikely | High |
| Aetiology of liver disease - Nonviral | 365 | | 0.61 [0.47, 0.80] | Low | No inconsistency | No indirectness | No imprecision | Unlikely | High |
| Barcelona Clinic liver cancer stage - A | 326 | | 0.78 [0.58, 1.03] | Low | No inconsistency | No indirectness | No imprecision | Unlikely | High |
| Barcelona Clinic liver cancer stage - B | 725 | | 0.68 [0.57, 0.82] | Low | No inconsistency | No indirectness | No imprecision | Unlikely | High |
| Barcelona Clinic liver cancer stage - C | 261 | | 0.66 [0.40, 1.09] | Low | Serious (-1) | No indirectness | No imprecision | Unlikely | Medium |
| Portal vein invasion - Vp1/Vp2 | 63 | | 0.70 [0.36, 1.36] | Low | No inconsistency | No indirectness | No imprecision | Unlikely | High |
| Portal vein invasion - None | 888 | | 0.59 [0.39, 0.88] | Low | Serious (-1) | No indirectness | No imprecision | Unlikely | Medium |
| Child-Pugh score at screening - Class A=5 | 694 | | 0.63 [0.52, 0.77] | Low | No inconsistency | No indirectness | No imprecision | Unlikely | High |
| Child-Pugh score at screening - Class A=6 | 128 | | 1.08 [0.71, 1.64] | Low | No inconsistency | No indirectness | No imprecision | Unlikely | High |
| Baseline tumour burden (criteria) - 6/7 or less | 448 | | 0.70 [0.55, 0.89] | Low | No inconsistency | No indirectness | No imprecision | Unlikely | High |
| Baseline tumour burden (criteria) - > 6/7 | 967 | | 0.58 [0.41, 0.82] | Low | Serious (-1) | No indirectness | No imprecision | Unlikely | Medium |
| Baseline albumin-bilirubin grade - 1 | 588 | | 0.65 [0.53, 0.80] | Low | No inconsistency | No indirectness | No imprecision | Unlikely | High |
| Baseline albumin-bilirubin grade - 2 or more | 300 | | 0.79 [0.60, 1.03] | Low | No inconsistency | No indirectness | No imprecision | Unlikely | High |
| PD-1/PD-L1 inhibitor type - Durvalumab | 409 | | 0.77 [0.61, 0.98] | Low | No inconsistency | No indirectness | No imprecision | Unlikely | High |
| PD-1/PD-L1 inhibitor type - Pembrolizumab | 480 | | 0.66 [0.51, 0.85] | Low | No inconsistency | No indirectness | No imprecision | Unlikely | High |
| PD-1/PD-L1 inhibitor type - Atezolizumab | 342 | | 0.71 [0.55, 0.92] | Low | No inconsistency | No indirectness | No imprecision | Unlikely | High |
| PD-1/PD-L1 inhibitor type - Camrelizumab | 200 | | 0.36 [0.25, 0.52] | Low | No inconsistency | No indirectness | No imprecision | Unlikely | High |
| Anti-VEGF drug type - Bevacizumab | 751 | | 0.74 [0.62, 0.88] | Low | No inconsistency | No indirectness | No imprecision | Unlikely | High |
| Anti-VEGF drug type - Lenvatinib | 480 | | 0.66 [0.51, 0.85] | Low | No inconsistency | No indirectness | No imprecision | Unlikely | High |
| Anti-VEGF drug type - Rivoceranib | 200 | | 0.36 [0.25, 0.52] | Low | No inconsistency | No indirectness | No imprecision | Unlikely | High |
| **Responses according to RECIST version 1.1** |  |  |  |  |  |  |  |  |  |
| ORR | 283/612 | 199/619 | 1.44 [1.25, 1.66] | Low | No inconsistency | No indirectness | No imprecision | Unlikely | High |
| DCR | 489/612 | 438/619 | 1.13 [1.06, 1.20] | Low | No inconsistency | No indirectness | No imprecision | Unlikely | High |
| CR | 20/612 | 22/619 | 0.92 [0.51, 1.67] | Low | No inconsistency | No indirectness | No imprecision | Unlikely | High |
| PR | 263/612 | 177/619 | 1.50 [1.29, 1.75] | Low | No inconsistency | No indirectness | No imprecision | Unlikely | High |
| SD | 196/612 | 239/619 | 0.83 [0.72, 0.97] | Low | No inconsistency | No indirectness | No imprecision | Unlikely | High |
| PD | 107/612 | 159/619 | 0.61 [0.38, 0.97] | Low | Serious (-1) | No indirectness | No imprecision | Unlikely | Medium |
| Duration of response | 541 | 548 | 3.69 [1.41, 5.97] | Low | Serious (-1) | No indirectness | No imprecision | Unlikely | Medium |
| **Responses according to mRECIST** |  |  |  |  |  |  |  |  |  |
| ORR | 369/508 | 264/514 | 1.47 [1.16, 1.87] | Low | Serious (-1) | No indirectness | No imprecision | Unlikely | Medium |
| DCR | 458/508 | 391/514 | 1.21 [1.07, 1.36] | Low | Serious (-1) | No indirectness | No imprecision | Unlikely | Medium |
| CR | 216/508 | 134/514 | 1.64 [1.38, 1.94] | Low | No inconsistency | No indirectness | No imprecision | Unlikely | High |
| PR | 153/508 | 130/514 | 1.22 [0.86, 1.72] | Low | Serious (-1) | No indirectness | No imprecision | Unlikely | Medium |
| SD | 89/508 | 127/514 | 0.71 [0.56, 0.90] | Low | No inconsistency | No indirectness | No imprecision | Unlikely | High |
| PD | 21/508 | 90/514 | 0.24 [0.15, 0.37] | Low | No inconsistency | No indirectness | No imprecision | Unlikely | High |
| **Patient status at cutoff time** |  |  |  |  |  |  |  |  |  |
| **Ongoing/Completed treatment** | 177/612 | 144/619 | 1.29 [0.84, 1.96] | Low | No inconsistency | No indirectness | No imprecision | Unlikely | High |
| **Exclusion** |  |  |  |  |  |  |  |  |  |
| Total exclusion | 424/612 | 469/619 | 0.92 [0.86, 0.98] | Low | No inconsistency | No indirectness | No imprecision | Unlikely | High |
| Exclusion due to progression | 250/612 | 352/619 | 0.74 [0.58, 0.94] | Low | Serious (-1) | No indirectness | No imprecision | Unlikely | Medium |
| Exclusion due to patient decision | 45/612 | 52/619 | 0.87 [0.60, 1.28] | Low | No inconsistency | No indirectness | No imprecision | Unlikely | High |
| Exclusion due to adverse events | 86/441 | 28/448 | 3.12 [2.08, 4.67] | Low | No inconsistency | No indirectness | No imprecision | Unlikely | High |
| **TEAEs** |  |  |  |  |  |  |  |  |  |
| Total TEAEs | 553/557 | 591/616 | 1.03 [0.99, 1.07] | Low | Serious (-1) | No indirectness | No imprecision | Unlikely | Medium |
| Grade 3-4 TEAEs | 378/557 | 249/616 | 1.63 [1.32, 2.02] | Low | Serious (-1) | No indirectness | No imprecision | Unlikely | Medium |
| Serious TEAEs | 141/320 | 103/373 | 1.62 [1.32, 1.98] | Low | No inconsistency | No indirectness | No imprecision | Unlikely | High |
| TEAEs leading to discontinuation | 157/557 | 37/616 | 4.73 [3.36, 6.65] | Low | No inconsistency | No indirectness | No imprecision | Unlikely | High |
| TEAEs leading to death | 33/557 | 22/616 | 1.73 [1.03, 2.93] | Low | No inconsistency | No indirectness | No imprecision | Unlikely | High |
| **TRAEs** |  |  |  |  |  |  |  |  |  |
| Total TRAEs | 524/557 | 463/616 | 1.28 [0.90, 1.83] | Low | Serious (-1) | No indirectness | No imprecision | Unlikely | Medium |
| Grade 3-4 TRAEs | 311/557 | 158/616 | 2.28 [1.45, 3.60] | Low | Serious (-1) | No indirectness | No imprecision | Unlikely | Medium |
| Serious TRAEs | 73/320 | 34/373 | 2.58 [1.26, 5.28] | Low | Serious (-1) | No indirectness | No imprecision | Unlikely | Medium |
| TRAEs leading to discontinuation | 83/391 | 15/443 | 6.11 [3.57, 10.47] | Low | No inconsistency | No indirectness | No imprecision | Unlikely | High |
| TRAEs leading to death | 9/557 | 7/616 | 1.39 [0.53, 3.69] | Low | No inconsistency | No indirectness | No imprecision | Unlikely | High |
| **irAEs** |  |  |  |  |  |  |  |  |  |
| Total irAEs | 165/391 | 49/443 | 3.76 [2.82, 5.02] | Low | No inconsistency | No indirectness | No imprecision | Unlikely | High |
| Grade 3-5 irAEs | 21/237 | 4/243 | 5.38 [1.88, 15.45] | Low | No inconsistency | No indirectness | No imprecision | Unlikely | High |
| **Any grade TRAEs** |  |  |  |  |  |  |  |  |  |
| Increased aspartate aminotransferase | 192/497 | 137/519 | 1.50 [1.12, 2.01] | Low | Serious (-1) | No indirectness | No imprecision | Unlikely | Medium |
| Hypertension | 234/651 | 69/719 | 3.81 [2.37, 6.14] | Low | Serious (-1) | No indirectness | No imprecision | Unlikely | Medium |
| Proteinuria | 234/651 | 28/719 | 12.99 [3.88, 43.53] | Low | Serious (-1) | No indirectness | No imprecision | Unlikely | Medium |
| Post-embolisation syndrome | 163/557 | 209/616 | 0.86 [0.72, 1.01] | Low | No inconsistency | No indirectness | No imprecision | Unlikely | High |
| Hypothyroidism | 112/391 | 38/443 | 3.18 [1.34, 7.55] | Low | Serious (-1) | No indirectness | No imprecision | Unlikely | Medium |
| Increased alanine aminotransferase | 181/651 | 144/719 | 1.33 [1.10, 1.59] | Low | No inconsistency | No indirectness | No imprecision | Unlikely | High |
| Palmar-plantar erythrodysesthesia syndrome | 88/331 | 4/346 | 20.46 [8.06, 51.95] | Low | No inconsistency | No indirectness | No imprecision | Unlikely | High |
| Decreased platelet count | 172/651 | 86/719 | 2.30 [1.48, 3.57] | Low | Serious (-1) | No indirectness | No imprecision | Unlikely | Medium |
| Hyperbilirubinaemia | 151/651 | 84/719 | 1.96 [1.16, 3.32] | Low | Serious (-1) | No indirectness | No imprecision | Unlikely | Medium |
| Pyrexia | 140/651 | 128/719 | 1.19 [0.97, 1.47] | Low | No inconsistency | No indirectness | No imprecision | Unlikely | High |
| Decreased appetite | 102/485 | 49/546 | 2.31 [1.12, 4.77] | Low | Serious (-1) | No indirectness | No imprecision | Unlikely | Medium |
| Hypoalbuminaemia | 103/497 | 67/519 | 1.61 [1.22, 2.13] | Low | No inconsistency | No indirectness | No imprecision | Unlikely | High |
| Diarrhoea | 100/485 | 47/546 | 3.64 [0.66, 19.93] | Low | Serious (-1) | No indirectness | No imprecision | Unlikely | Medium |
| Abdominal pain upper | 121/651 | 121/719 | 1.09 [0.88, 1.36] | Low | No inconsistency | No indirectness | No imprecision | Unlikely | High |
| Decreased white blood cell count | 60/331 | 20/346 | 6.36 [0.29, 140.39] | Low | Serious (-1) | No indirectness | No imprecision | Unlikely | Medium |
| Fatigue | 86/485 | 53/546 | 1.80 [1.31, 2.49] | Low | No inconsistency | No indirectness | No imprecision | Unlikely | High |
| Abdominal pain | 69/391 | 72/443 | 1.08 [0.80, 1.45] | Low | No inconsistency | No indirectness | No imprecision | Unlikely | High |
| Decreased neutrophil count | 52/331 | 14/346 | 6.54 [0.46, 93.10] | Low | Serious (-1) | No indirectness | No imprecision | Unlikely | Medium |
| Nausea | 66/485 | 50/546 | 1.46 [1.03, 2.07] | Low | No inconsistency | No indirectness | No imprecision | Unlikely | High |
| Increased γ-glutamyltransferase | 45/331 | 23/346 | 2.04 [1.26, 3.30] | Low | No inconsistency | No indirectness | No imprecision | Unlikely | High |
| Pruritus | 50/391 | 33/443 | 1.82 [1.20, 2.74] | Low | No inconsistency | No indirectness | No imprecision | Unlikely | High |
| Anemia | 61/485 | 40/546 | 1.74 [1.19, 2.55] | Low | No inconsistency | No indirectness | No imprecision | Unlikely | High |
| Rash | 48/391 | 10/443 | 6.12 [1.34, 27.93] | Low | Serious (-1) | No indirectness | No imprecision | Unlikely | Medium |
| Constipation | 59/485 | 44/546 | 1.56 [1.08, 2.25] | Low | No inconsistency | No indirectness | No imprecision | Unlikely | High |
| Vomiting | 39/331 | 25/346 | 1.81 [0.71, 4.61] | Low | No inconsistency | No indirectness | No imprecision | Unlikely | High |
| Arthralgia | 37/391 | 33/443 | 1.33 [0.85, 2.08] | Low | No inconsistency | No indirectness | No imprecision | Unlikely | High |
| Hypokalaemia | 29/331 | 22/346 | 1.87 [0.35, 10.02] | Low | Serious (-1) | No indirectness | No imprecision | Unlikely | Medium |
| Increased blood alkaline phosphatase | 24/331 | 15/346 | 1.68 [0.90, 3.14] | Low | No inconsistency | No indirectness | No imprecision | Unlikely | High |
| Pneumonia | 5/154 | 2/200 | 3.25 [0.64, 16.51] | Low | No inconsistency | No indirectness | No imprecision | Unlikely | High |
| **Grade 3-5 TRAEs** |  |  |  |  |  |  |  |  |  |
| Hypertension | 79/485 | 19/546 | 4.29 [2.68, 6.86] | Low | No inconsistency | No indirectness | No imprecision | Unlikely | High |
| Increased aspartate aminotransferase | 45/331 | 26/346 | 1.84 [1.18, 2.89] | Low | No inconsistency | No indirectness | No imprecision | Unlikely | High |
| Decreased platelet count | 41/485 | 19/546 | 2.28 [1.35, 3.84] | Low | No inconsistency | No indirectness | No imprecision | Unlikely | High |
| Increased alanine aminotransferase | 36/485 | 26/546 | 1.50 [0.93, 2.40] | Low | No inconsistency | No indirectness | No imprecision | Unlikely | High |
| Diarrhoea | 25/485 | 0/546 | 19.53 [3.71, 102.81] | Low | No inconsistency | No indirectness | No imprecision | Unlikely | High |
| Palmar-plantar erythrodysesthesia syndrome | 16/331 | 0/346 | 17.87 [2.37, 134.62] | Low | No inconsistency | No indirectness | No imprecision | Unlikely | High |
| Increased γ-glutamyltransferase | 16/331 | 2/346 | 6.90 [1.85, 25.76] | Low | No inconsistency | No indirectness | No imprecision | Unlikely | High |
| Proteinuria | 18/485 | 0/546 | 14.63 [2.78, 76.97] | Low | No inconsistency | No indirectness | No imprecision | Unlikely | High |
| Decreased neutrophil count | 12/331 | 4/346 | 2.89 [1.00, 8.37] | Low | No inconsistency | No indirectness | No imprecision | Unlikely | High |
| Hyperbilirubinaemia | 16/485 | 5/546 | 3.21 [1.24, 8.31] | Low | No inconsistency | No indirectness | No imprecision | Unlikely | High |
| Decreased white blood cell count | 10/331 | 1/346 | 7.31 [1.33, 40.30] | Low | No inconsistency | No indirectness | No imprecision | Unlikely | High |
| Hypokalaemia | 10/331 | 6/346 | 1.74 [0.64, 4.72] | Low | No inconsistency | No indirectness | No imprecision | Unlikely | High |
| Fatigue | 13/485 | 4/546 | 3.58 [1.18, 10.92] | Low | No inconsistency | No indirectness | No imprecision | Unlikely | High |
| Anemia | 13/485 | 5/546 | 3.09 [1.11, 8.61] | Low | No inconsistency | No indirectness | No imprecision | Unlikely | High |
| Post-embolisation syndrome | 8/391 | 13/443 | 0.73 [0.31, 1.74] | Low | No inconsistency | No indirectness | No imprecision | Unlikely | High |
| Pneumonia | 6/391 | 1/443 | 4.91 [0.86, 28.14] | Low | No inconsistency | No indirectness | No imprecision | Unlikely | High |
| Decreased appetite | 5/485 | 3/546 | 1.65 [0.46, 5.91] | Low | No inconsistency | No indirectness | No imprecision | Unlikely | High |
| Abdominal pain | 4/391 | 3/443 | 1.43 [0.37, 5.54] | Low | No inconsistency | No indirectness | No imprecision | Unlikely | High |
| Rash | 4/391 | 0/443 | 5.64 [0.65, 49.17] | Low | No inconsistency | No indirectness | No imprecision | Unlikely | High |
| Increased blood alkaline phosphatase | 3/331 | 0/346 | 4.25 [0.48, 37.62] | Low | No inconsistency | No indirectness | No imprecision | Unlikely | High |
| Pyrexia | 4/485 | 2/546 | 2.10 [0.46, 9.50] | Low | No inconsistency | No indirectness | No imprecision | Unlikely | High |
| Abdominal pain upper | 3/485 | 4/546 | 0.82 [0.19, 3.58] | Low | No inconsistency | No indirectness | No imprecision | Unlikely | High |
| Hypoalbuminaemia | 1/331 | 0/346 | 3.08 [0.13, 75.12] | Low | No inconsistency | No indirectness | No imprecision | Unlikely | High |
| Hypothyroidism | 1/391 | 0/443 | 3.08 [0.13, 75.12] | Low | No inconsistency | No indirectness | No imprecision | Unlikely | High |
| Constipation | 1/485 | 0/546 | 3.08 [0.13, 75.12] | Low | No inconsistency | No indirectness | No imprecision | Unlikely | High |
| Nausea | 1/485 | 0/546 | 3.08 [0.13, 75.12] | Low | No inconsistency | No indirectness | No imprecision | Unlikely | High |
| Pruritus | 0/391 | 1/443 | 0.34 [0.01, 8.35] | Low | No inconsistency | No indirectness | No imprecision | Unlikely | High |
| Vomiting | 0/331 | 1/346 | 0.36 [0.02, 8.85] | Low | No inconsistency | No indirectness | No imprecision | Unlikely | High |

**Abbreviations:** AEs: Adverse events; ALT: Alanine aminotransferase; AST: Aspartate aminotransferase; AVDs: Anti-VEGF drugs; BCLC: Barcelona Clinic Liver Cancer; CI: Confidence interval; CR: Complete response; DCR: Disease control rate; ECOG PS: Eastern Cooperative Oncology Group Performance Status; GRADE: Grading of Recommendations Assessment, Development and Evaluation; HR: Hazard ratio; irAEs: Immune-related adverse events; MD: Mean difference; M/F: Male/Female; mRECIST: Modified Response Evaluation Criteria in Solid Tumors; ORR: Objective response rate; OS: Overall survival; OSR: Overall survival rate; P: Probability; PD: Progressive disease; PD-1: Programmed cell death protein 1; PD-L1: Programmed death-ligand 1; PFS: Progression-free survival; PFSR: Progression-free survival rate; PIs: PD-1/PD-L1 inhibitors; PR: Partial response; RCT: Randomized controlled trial; RECIST: Response Evaluation Criteria in Solid Tumors; RR: Risk ratio; SD: Stable disease; TACE: Transarterial chemoembolization; TEAEs: Treatment-emergent adverse events; TPA: TACE plus PIs and AVDs; TRAEs: Treatment-related adverse events; TTP: Time to progression; VEGF: Vascular endothelial growth factor.

^a^ Differences: HR for PFS, OS, and TTP; RR for PFSR, OSR, responses, and AEs; MD for duration of response.

^b^ Risk of bias assessed using the Jadad scale for randomized controlled trials.

^c^ Publication bias was explored through visual inspection of the funnel plots.
