# Supplementary material for: The benefit and risk of adding PD-1/PD-L1 inhibitors plus anti-VEGF drugs to transarterial chemoembolisation for unresectable, non-metastatic hepatocellular carcinoma: a pooled analysis of four RCTs
Source: Front Med (Lausanne). 2026 May 25;13:1792746. doi: 10.3389/fmed.2026.1792746 (PMC13244568; doi:10.3389/fmed.2026.1792746)
Supplement: Supplementary file 11 [file Table_4.doc]

**Table S4** Patient status at cutoff time.

| **Patient status** | **TPA** | |  | **TACE** | | **Risk ratio [95% CI]** | ***I^2^*** | **P** |
| --- | --- | --- | --- | --- | --- | --- | --- | --- |
|  | **Event/total** | **%** |  | **Event/total** | **%** |  |  |  |
| **Ongoing/Completed treatment** | 177/612 | 28.92% |  | 144/619 | 23.26% | 1.29 [0.84, 1.96] | 75% | 0.24 |
| **Exclusion** |  |  |  |  |  |  |  |  |
| Total exclusion | 424/612 | 69.28% |  | 469/619 | 75.77% | 0.92 [0.86, 0.98] | 6% | 0.007 |
| Exclusion due to progression | 250/612 | 40.85% |  | 352/619 | 56.87% | 0.74 [0.58, 0.94] | 74% | 0.01 |
| Exclusion due to patient decision | 45/612 | 7.35% |  | 52/619 | 8.40% | 0.87 [0.60, 1.28] | 22% | 0.49 |
| Exclusion due to adverse events | 86/441 | 19.50% |  | 28/448 | 6.25% | 3.12 [2.08, 4.67] | 0% | < 0.00001 |

**Abbreviations:** AVDs: Anti-VEGF drugs; CI: Confidence interval; *I²*: I-squared statistic; P: Probability; PD-1: Programmed cell death protein 1; PD-L1: Programmed death-ligand 1; PIs: PD-1/PD-L1 inhibitors; RR: Risk ratio; TACE: Transarterial chemoembolization; TPA: TACE plus PIs and AVDs; VEGF: Vascular endothelial growth factor.
