# Supplementary material for: The benefit and risk of adding PD-1/PD-L1 inhibitors plus anti-VEGF drugs to transarterial chemoembolisation for unresectable, non-metastatic hepatocellular carcinoma: a pooled analysis of four RCTs
Source: Front Med (Lausanne). 2026 May 25;13:1792746. doi: 10.3389/fmed.2026.1792746 (PMC13244568; doi:10.3389/fmed.2026.1792746)
Supplement: Supplementary file 12 [file Table_5.doc]

**Table S5** Any grade and grade 3-5 treatment-related adverse events (all).

| **TRAEs** | **TPA** | |  | **TACE** | | **Risk ratio [95% CI]** | ***I^2^*** | **P** |
| --- | --- | --- | --- | --- | --- | --- | --- | --- |
|  | **Event/total** | **%** |  | **Event/total** | **%** |  |  |  |
| **Any grade TRAEs** |  |  |  |  |  |  |  |  |
| Increased aspartate aminotransferase | 192/497 | 38.63% |  | 137/519 | 26.40% | 1.50 [1.12, 2.01] | 61% | 0.006 |
| Hypertension | 234/651 | 35.94% |  | 69/719 | 9.60% | 3.81 [2.37, 6.14] | 60% | < 0.00001 |
| Proteinuria | 234/651 | 35.94% |  | 28/719 | 3.89% | 12.99 [3.88, 43.53] | 80% | < 0.0001 |
| Post-embolisation syndrome | 163/557 | 29.26% |  | 209/616 | 33.93% | 0.86 [0.72, 1.01] | 0% | 0.07 |
| Hypothyroidism | 112/391 | 28.64% |  | 38/443 | 8.58% | 3.18 [1.34, 7.55] | 83% | 0.009 |
| Increased alanine aminotransferase | 181/651 | 27.80% |  | 144/719 | 20.03% | 1.33 [1.10, 1.59] | 44% | 0.002 |
| Palmar-plantar erythrodysesthesia syndrome | 88/331 | 26.59% |  | 4/346 | 1.16% | 20.46 [8.06, 51.95] | 1% | < 0.00001 |
| Decreased platelet count | 172/651 | 26.42% |  | 86/719 | 11.96% | 2.30 [1.48, 3.57] | 61% | 0.0002 |
| Hyperbilirubinaemia | 151/651 | 23.20% |  | 84/719 | 11.68% | 1.96 [1.16, 3.32] | 70% | 0.01 |
| Pyrexia | 140/651 | 21.51% |  | 128/719 | 17.80% | 1.19 [0.97, 1.47] | 0% | 0.10 |
| Decreased appetite | 102/485 | 21.03% |  | 49/546 | 8.97% | 2.31 [1.12, 4.77] | 75% | 0.02 |
| Hypoalbuminaemia | 103/497 | 20.72% |  | 67/519 | 12.91% | 1.61 [1.22, 2.13] | 57% | 0.0009 |
| Diarrhoea | 100/485 | 20.62% |  | 47/546 | 8.61% | 3.64 [0.66, 19.93] | 93% | 0.14 |
| Abdominal pain upper | 121/651 | 18.59% |  | 121/719 | 16.83% | 1.09 [0.88, 1.36] | 43% | 0.42 |
| Decreased white blood cell count | 60/331 | 18.13% |  | 20/346 | 5.78% | 6.36 [0.29, 140.39] | 89% | 0.24 |
| Fatigue | 86/485 | 17.73% |  | 53/546 | 9.71% | 1.80 [1.31, 2.49] | 51% | 0.0003 |
| Abdominal pain | 69/391 | 17.65% |  | 72/443 | 16.25% | 1.08 [0.80, 1.45] | 26% | 0.63 |
| Decreased neutrophil count | 52/331 | 15.71% |  | 14/346 | 4.05% | 6.54 [0.46, 93.10] | 85% | 0.17 |
| Nausea | 66/485 | 13.61% |  | 50/546 | 9.16% | 1.46 [1.03, 2.07] | 24% | 0.03 |
| Increased γ-glutamyltransferase | 45/331 | 13.60% |  | 23/346 | 6.65% | 2.04 [1.26, 3.30] | 0% | 0.004 |
| Pruritus | 50/391 | 12.79% |  | 33/443 | 7.45% | 1.82 [1.20, 2.74] | 0% | 0.005 |
| Anemia | 61/485 | 12.58% |  | 40/546 | 7.33% | 1.74 [1.19, 2.55] | 54% | 0.004 |
| Rash | 48/391 | 12.28% |  | 10/443 | 2.26% | 6.12 [1.34, 27.93] | 72% | 0.02 |
| Constipation | 59/485 | 12.16% |  | 44/546 | 8.06% | 1.56 [1.08, 2.25] | 0% | 0.02 |
| Vomiting | 39/331 | 11.78% |  | 25/346 | 7.23% | 1.81 [0.71, 4.61] | 68% | 0.22 |
| Arthralgia | 37/391 | 9.46% |  | 33/443 | 7.45% | 1.33 [0.85, 2.08] | 0% | 0.21 |
| Hypokalaemia | 29/331 | 8.76% |  | 22/346 | 6.36% | 1.87 [0.35, 10.02] | 83% | 0.47 |
| Increased blood alkaline phosphatase | 24/331 | 7.25% |  | 15/346 | 4.34% | 1.68 [0.90, 3.14] | 0% | 0.10 |
| Pneumonia | 5/154 | 3.25% |  | 2/200 | 1.00% | 3.25 [0.64, 16.51] | - | 0.16 |
| **Grade 3-4 TRAEs** |  |  |  |  |  |  |  |  |
| Hypertension | 79/485 | 16.29% |  | 19/546 | 3.48% | 4.29 [2.68, 6.86] | 49% | < 0.00001 |
| Increased aspartate aminotransferase | 45/331 | 13.60% |  | 26/346 | 7.51% | 1.84 [1.18, 2.89] | 49% | 0.008 |
| Decreased platelet count | 41/485 | 8.45% |  | 19/546 | 3.48% | 2.28 [1.35, 3.84] | 0% | 0.002 |
| Increased alanine aminotransferase | 36/485 | 7.42% |  | 26/546 | 4.76% | 1.50 [0.93, 2.40] | 0% | 0.09 |
| Diarrhoea | 25/485 | 5.15% |  | 0/546 | 0.00% | 19.53 [3.71, 102.81] | 0% | 0.0005 |
| Palmar-plantar erythrodysesthesia syndrome | 16/331 | 4.83% |  | 0/346 | 0.00% | 17.87 [2.37, 134.62] | 0% | 0.005 |
| Increased γ-glutamyltransferase | 16/331 | 4.83% |  | 2/346 | 0.58% | 6.90 [1.85, 25.76] | 0% | 0.004 |
| Proteinuria | 18/485 | 3.71% |  | 0/546 | 0.00% | 14.63 [2.78, 76.97] | 0% | 0.002 |
| Decreased neutrophil count | 12/331 | 3.63% |  | 4/346 | 1.16% | 2.89 [1.00, 8.37] | 0% | 0.05 |
| Hyperbilirubinaemia | 16/485 | 3.30% |  | 5/546 | 0.92% | 3.21 [1.24, 8.31] | 0% | 0.02 |
| Decreased white blood cell count | 10/331 | 3.02% |  | 1/346 | 0.29% | 7.31 [1.33, 40.30] | 0% | 0.02 |
| Hypokalaemia | 10/331 | 3.02% |  | 6/346 | 1.73% | 1.74 [0.64, 4.72] | 3% | 0.28 |
| Fatigue | 13/485 | 2.68% |  | 4/546 | 0.73% | 3.58 [1.18, 10.92] | 0% | 0.02 |
| Anemia | 13/485 | 2.68% |  | 5/546 | 0.92% | 3.09 [1.11, 8.61] | 0% | 0.03 |
| Post-embolisation syndrome | 8/391 | 2.05% |  | 13/443 | 2.93% | 0.73 [0.31, 1.74] | 0% | 0.48 |
| Pneumonia | 6/391 | 1.53% |  | 1/443 | 0.23% | 4.91 [0.86, 28.14] | 0% | 0.07 |
| Decreased appetite | 5/485 | 1.03% |  | 3/546 | 0.55% | 1.65 [0.46, 5.91] | 0% | 0.44 |
| Abdominal pain | 4/391 | 1.02% |  | 3/443 | 0.68% | 1.43 [0.37, 5.54] | 38% | 0.61 |
| Rash | 4/391 | 1.02% |  | 0/443 | 0.00% | 5.64 [0.65, 49.17] | 0% | 0.12 |
| Increased blood alkaline phosphatase | 3/331 | 0.91% |  | 0/346 | 0.00% | 4.25 [0.48, 37.62] | 0% | 0.19 |
| Pyrexia | 4/485 | 0.82% |  | 2/546 | 0.37% | 2.10 [0.46, 9.50] | 0% | 0.34 |
| Abdominal pain upper | 3/485 | 0.62% |  | 4/546 | 0.73% | 0.82 [0.19, 3.58] | - | 0.79 |
| Hypoalbuminaemia | 1/331 | 0.30% |  | 0/346 | 0.00% | 3.08 [0.13, 75.12] | - | 0.49 |
| Hypothyroidism | 1/391 | 0.26% |  | 0/443 | 0.00% | 3.08 [0.13, 75.12] | - | 0.49 |
| Constipation | 1/485 | 0.21% |  | 0/546 | 0.00% | 3.08 [0.13, 75.12] | - | 0.49 |
| Nausea | 1/485 | 0.21% |  | 0/546 | 0.00% | 3.08 [0.13, 75.12] | - | 0.49 |
| Pruritus | 0/391 | 0.00% |  | 1/443 | 0.23% | 0.34 [0.01, 8.35] | - | 0.51 |
| Vomiting | 0/331 | 0.00% |  | 1/346 | 0.29% | 0.36 [0.02, 8.85] | - | 0.54 |

**Abbreviations:** AVDs: Anti-VEGF drugs; CI: Confidence interval; *I²*: I-squared statistic; P: Probability; PD-1: Programmed cell death protein 1; PD-L1: Programmed death-ligand 1; PIs: PD-1/PD-L1 inhibitors; RR: Risk ratio; TACE: Transarterial chemoembolization; TPA: TACE plus PIs and AVDs; TRAEs: Treatment-related adverse events; VEGF: Vascular endothelial growth factor.
